# Ethnic Variation in Inflammatory Profile in Tuberculosis

**DOI:** 10.1371/journal.ppat.1003468

**Published:** 2013-07-04

**Authors:** Anna K. Coussens, Robert J. Wilkinson, Vladyslav Nikolayevskyy, Paul T. Elkington, Yasmeen Hanifa, Kamrul Islam, Peter M. Timms, Graham H. Bothamley, Alleyna P. Claxton, Geoffrey E. Packe, Mathina Darmalingam, Robert N. Davidson, Heather J. Milburn, Lucy V. Baker, Richard D. Barker, Francis A. Drobniewski, Charles A. Mein, Leena Bhaw-Rosun, Rosamond A. Nuamah, Christopher J. Griffiths, Adrian R. Martineau

**Affiliations:** 1 Division of Mycobacterial Research, MRC National Institute for Medical Research, London, United Kingdom; 2 Division of Medicine, Imperial College London, London, United Kingdom; 3 Queen Mary University of London, Barts and The London School of Medicine and Dentistry, Blizard Institute, London, United Kingdom; 4 Department of Infectious Diseases and Immunity, Imperial College, London, United Kingdom; 5 Homerton University NHS Foundation Trust, London, United Kingdom; 6 Newham Chest Clinic, Forest Gate, London, United Kingdom; 7 Department of Respiratory Medicine, Whipps Cross University Hospital, London, United Kingdom; 8 Tuberculosis Clinic, Northwick Park Hospital, Harrow, United Kingdom; 9 Department of Respiratory Medicine, Guy's and St Thomas' NHS Foundation Trust, London, United Kingdom; 10 Department of Respiratory Medicine, Lewisham Hospital, London, United Kingdom; 11 Department of Respiratory Medicine, Kings College Hospital, London, United Kingdom; 12 Genome Centre, Queen Mary University of London, Barts and The London School of Medicine, London, United Kingdom; University of Washington, United States of America

## Abstract

Distinct phylogenetic lineages of *Mycobacterium tuberculosis* (MTB) cause disease in patients of particular genetic ancestry, and elicit different patterns of cytokine and chemokine secretion when cultured with human macrophages *in vitro*. Circulating and antigen-stimulated concentrations of these inflammatory mediators might therefore be expected to vary significantly between tuberculosis patients of different ethnic origin. Studies to characterise such variation, and to determine whether it relates to host or bacillary factors, have not been conducted. We therefore compared circulating and antigen-stimulated concentrations of 43 inflammatory mediators and 14 haematological parameters (inflammatory profile) in 45 pulmonary tuberculosis patients of African ancestry vs. 83 patients of Eurasian ancestry in London, UK, and investigated the influence of bacillary and host genotype on these profiles. Despite having similar demographic and clinical characteristics, patients of differing ancestry exhibited distinct inflammatory profiles at presentation: those of African ancestry had lower neutrophil counts, lower serum concentrations of CCL2, CCL11 and vitamin D binding protein (DBP) but higher serum CCL5 concentrations and higher antigen-stimulated IL-1 receptor antagonist and IL-12 secretion. These differences associated with ethnic variation in host *DBP* genotype, but not with ethnic variation in MTB strain. Ethnic differences in inflammatory profile became more marked following initiation of antimicrobial therapy, and immunological correlates of speed of elimination of MTB from the sputum differed between patients of African vs. Eurasian ancestry. Our study demonstrates a hitherto unappreciated degree of ethnic heterogeneity in inflammatory profile in tuberculosis patients that associates primarily with ethnic variation in host, rather than bacillary, genotype. Candidate immunodiagnostics and immunological biomarkers of response to antimicrobial therapy should be derived and validated in tuberculosis patients of different ethnic origin.

## Author Summary

*Mycobacterium tuberculosis* (MTB) is the causative agent of tuberculosis. Genetically distinct strains of MTB cause disease in particular ethnic groups, and these strains vary in their ability to elicit inflammatory responses from antigen-presenting cells *in vitro*. Circulating and antigen-stimulated concentrations of inflammatory mediators (‘inflammatory profile’) might therefore be expected to differ between tuberculosis patients of different ethnic origin; however, this question has not previously been addressed. We therefore conducted a study to characterise ethnic variation in inflammatory profiles in a cohort of 128 newly-diagnosed tuberculosis patients in London, UK. Patients of African vs. Eurasian ancestry had distinct inflammatory profiles at presentation; differences did not relate to MTB strain variation between groups, but they did associate with ethnic variation in host genotype. Moreover, immunological correlates of the rate of MTB clearance from sputum differed between patients of African vs. Eurasian ancestry. Our findings provide insight into the mechanisms underlying ethnic variation in inflammatory profile in tuberculosis patients, and indicate that candidate immunodiagnostics and immunological biomarkers of response to tuberculosis therapy should be derived and validated in tuberculosis patients of different ethnic origin.

## Introduction

*Mycobacterium tuberculosis* (MTB), the causative agent of tuberculosis (TB), emerged as a pathogen in Africa and has co-evolved with humans following migration to Europe and Asia some 70,000 years ago [1]. Distinct phylogenetic lineages of MTB consistently associate with human populations of different genetic ancestry in a variety of settings [2]–[5] and elicit differing immune responses from antigen-presenting cells of healthy donors *in vitro* [6]–[11]. Antimycobacterial immune responses might therefore be expected to vary between TB patients of different ethnic origin; however, studies investigating this question have not been conducted. Demonstration of significant ethnic variation in inflammatory responses at presentation and after initiation of treatment would have implications for the development of immunodiagnostics and for the identification of surrogate endpoints for trials of antituberculous drugs.

We therefore conducted a study to characterise ethnic variation in circulating and antigen-stimulated concentrations of a panel of 43 soluble inflammatory mediators and 14 haematological parameters (collectively termed ‘inflammatory profile’) before and after intensive-phase antituberculous therapy in a multi-ethnic cohort of patients with pulmonary tuberculosis (PTB) who participated in a clinical trial of adjunctive vitamin D supplementation conducted in London, UK [12]. The primary comparison was between patients of African vs. Eurasian ancestry, on the grounds of the distinct genetic structure of these populations [13], and because TB patients of African ancestry are recognised to have delayed clearance of MTB from the sputum in comparison to non-African patients [14], [15] – a phenomenon that might be immunologically mediated_ENREF_17. We found that patients of African and Eurasian ancestry had significantly different inflammatory profiles at presentation, and that these differences associated primarily with variation in host, but not bacillary, genotype. Ethnic differences in inflammatory profile became more marked after intensive-phase treatment, and immunological correlates of time to sputum culture conversion between patients of African vs. Eurasian ancestry were distinct.

## Results

### PTB patients of African vs. Eurasian ancestry have distinct inflammatory profiles at presentation

A total of 141 patients were eligible to participate in the study (Study Profile, Figure S1). Self-defined ethnic origin was used to attribute ancestry as African (n = 45), Eurasian (n = 83), East Asian (n = 9), Latin American (n = 3) or mixed (n = 1) according to Rosenberg's five-region classification [13]. Due to small numbers in other groups, analyses were confined to participants of African and Eurasian ancestry. These two groups had similar demographic and clinical characteristics at presentation, the only statistically significant difference being a slightly shorter duration of symptoms pre-diagnosis in patients of African vs. Eurasian ancestry (median 2.0 vs. 2.5 months respectively, p = 0.03; Table 1).

**Table 1:** Baseline characteristics of tuberculosis patients of African vs. Eurasian ancestry.

	African ancestry (n = 45)	Eurasian ancestry (n = 83)	p
Median age, years (IQR)	29.3 (23.7 to 38.2)	30.8 (24.8 to 41.0)	0.43
Sex			
Male	31 (69)	65 (78)	0.24
Female	14 (31)	18 (22)	
BMI, kg/m^2^	19.9 (2.6)	20.1 (3.1)	0.73
Educated beyond 18 years	25 (56)	35 (42)	0.15
In UK before age 18 years	19 (42)	28 (34)	0.35
Occupation			
Student	9 (20)	9 (11)	0.28
Employed	32 (71)	62 (75)	
Unemployed	4 (9)	12 (14)	
Diabetes mellitus	2 (4)	5 (6)	>0.99
Median duration of symptoms pre-diagnosis, months (IQR)	2.0 (1.0 to 3.0)	2.5 (1.5 to 4.0)	0.03
Median duration of treatment pre-enrolment, days (IQR)	2.0 (0.0 to 4.0)	2.0 (0.0 to 3.0)	0.70
Baseline sputum smear^A^			
≤3 AFB per high-power field	22 (49)	37 (45)	0.73
>3 AFB per high-power field	23 (51)	44 (53)	
Median serum 25(OH)D, nmol/L (IQR)	18.0 (10.0 to 26.0)	16.0 (11.0 to 25.0)	0.73
Baseline chest radiograph			
Cavities present	22 (49)	47 (57)	0.40
Median no. zones affected, IQR	2.5 (2.0 to 4.0)	3.0 (2.0 to 4.0)	0.83
*DBP* genotype			
Gc1F/1F	22 (49)	5 (6)	<0.001
Gc1F/2	8 (18)	12 (14)	
Gc2/2	0 (0)	4 (5)	
Gc1F/1S	9 (20)	14 (17)	
Gc2/1S	1 (2)	27 (33)	
Gc1S/1S	5 (11)	21 (25)	
*M. tuberculosis* antimicrobial sensitivity			
Isoniazid-sensitive	43 (96)	72 (87)	0.14
Isoniazid-resistant	2 (4)	11 (13)	
*M. tuberculosis* strain lineage^B^			
Indo-Oceanic lineage	3 (7)	10 (12)	0.08
East Asian lineage	0 (0)	6 (7)	
East African-Indian lineage	12 (27)	27 (33)	
Euro-American lineage	29 (64)	39 (47)	
West African lineages	1 (2)	0 (0)	

Data are number (%) or mean (standard deviation) except where stated. IQR, inter-quartile range; BMI, body mass index; AFB, acid-fast bacilli. DBP, vitamin D binding protein; A, Baseline sputum smear result unavailable for 2 Eurasian participants; B, *M. tuberculosis* genotype unavailable for one Eurasian participant.

Forty-three soluble factors and 14 haematological parameters detailed in Table S1 were measured in samples of serum, plasma or whole blood taken at baseline. The median circulating concentrations of 7 soluble factors (Interleukin [IL]-2, IL-5, IL-13, IL-17, tumour necrosis factor [TNF], basic fibroblast growth factor [FGF-β] and matrix metalloproteinase-7 [MMP-7]) were below the limit of detection (LOD) at baseline and were excluded from further analyses; median values, ranges and LODs for these analytes at baseline are presented in Table S2.

**Figure 1:**
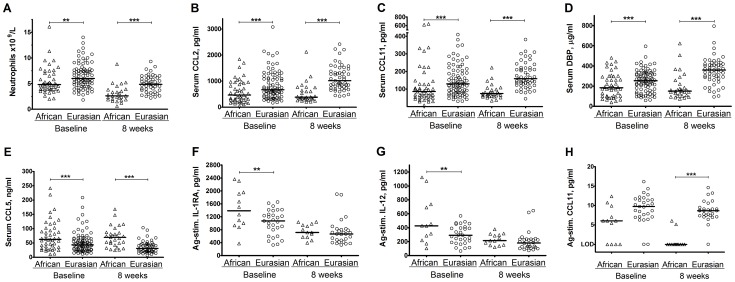
Ethnic variation in inflammatory profile in PTB before and after intensive-phase antituberculous therapy. At diagnosis, patients of African ancestry had lower neutrophil counts (A), lower serum concentrations of CCL2 (B), CCL11 (C) and DBP (D), but higher serum concentrations of CCL5 (E) and higher antigen-stimulated IL-1RA and IL-12 (F,G) than those of Eurasian ancestry (p≤0.0030). These differences persisted after completion of intensive-phase antimicrobial therapy for all circulating parameters illustrated (A–E; p≤7.84×10^−6^). 8-week antigen-stimulated CCL11 concentrations were also lower in patients of African vs. Eurasian ancestry (H; p = 6.45×10^−9^). P-values were derived from the t-test for general linear models, separately applied to baseline and 8-week samples, with adjustment for the following covariates: age, sex, months of symptoms pre-diagnosis, duration of antimicrobial therapy pre-sampling and either baseline vitamin D status (baseline samples) or isoniazid sensitivity and allocation to vitamin D vs. placebo (8-week samples). P-values are only indicated for parameters with a false discovery rate (q-value)≤0.05, as determined by the Benjamini Hochberg approach. *** p<0.001, ** p = 0.001 to <0.01. Lines at median. LOD, limit of detection.

**Figure 2:**
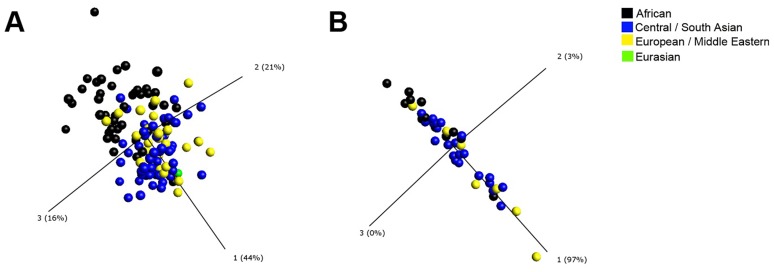
Principal component analysis (PCA) plots generated using immunological parameters which contribute to variation in baseline inflammatory profile between PTB patients of African and Eurasian ancestry. Each point represents one patient, and its position in the plot is determined by the combined effects of all parameters measured for that patient sample that contribute significantly to ethnic variation in inflammatory profile. The distance between sample points represents Euclidean distance. The first 3 component vectors are displayed, along with a % figure signifying the proportion of the variability in the data that each component accounts for. Points representing patients with Eurasian ancestry have been coloured according to their ethnic subgroup (Central/South Asian (blue) and European/Middle Eastern (yellow) ancestry) to demonstrate that they cluster together and that they are separated from those representing patients of African ancestry (black). One patient of mixed South Asian and European ancestry is classified as a Eurasian (green) and clusters within samples from the Eurasian subgroups. A, PCA plot of circulating immunological parameters in patients of African (n = 45), Central/South Asian (n = 55), European/Middle Eastern (n = 27) and Eurasian (n = 1) ancestry at baseline. B, PCA plot of rCFP-10-stimulated immunological parameters in patients of African (n = 13), Central/South Asian (n = 22) and European/Middle Eastern (n = 7) ancestry at baseline.

The remaining 50 parameters were then analysed using the t-test for general linear models (GLM) with statistical adjustment for covariates with potential to influence inflammatory profile (age, sex, duration of symptoms pre-diagnosis, duration of antimicrobial therapy pre-sampling and baseline serum 25-hydroxyvitamin D [25(OH)D] concentration). Five parameters were identified as having concentrations which were significantly different (false discovery rate [q-value]≤0.05) between participants of African vs. Eurasian ancestry. Four (peripheral blood neutrophil count and serum concentrations of CC chemokine ligand [CCL] 2, CCL11 and vitamin D binding protein [DBP]) were lower in participants of African vs. Eurasian ancestry (p≤0.0018), and one (serum CCL5 concentration) was higher (p = 2.15×10^−5^; Table 2; Figure 1). These parameters were then assessed using principal component analysis (PCA), a well-established mathematical technique for reducing the dimensionality of complex datasets by transforming the data to a new coordinate system [16]. This provided a visual representation of how well the identified parameters differentiated individuals from the two ethnic groups (Figure 2).

**Table 2:** Differences in inflammatory profile in PTB patients of African vs. Eurasian ancestry.

		Baseline						8 weeks	
		Adjusted for phenotypic characteristics only^A^		Adjusted for phenotypic characteristics^A^+MTB genotype^B^		Adjusted for phenotypic characteristics^A^+host DBP genotype^C^		Adjusted for phenotypic characteristics only^D^	
		t^E^	p^F^	t^E^	p^F^	t^E^	p^F^	t^E^	p^F^
Circulating	CCL2	−6.88	2.87×10^−10^	−6.76	5.96×10^−10^	−6.33	4.78×10^−09^	−7.13	5.67×10^−10^
	CCL5	4.42	2.15×10^−5^	4.35	2.98×10^−05^	3.33	0.0012	4.81	7.84×10^−06^
	CCL11	−7.84	2.00×10^−12^	−7.63	7.35×10^−12^	−6.79	5.15×10^−10^	−7.20	4.12×10^−10^
	CXCL8	-	ns	-	ns	-	ns	−3.23	0.0018
	DBP	−6.30	4.95×10^−09^	−5.99	2.43×10^−08^	-	ns	−5.93	8.94×10^−08^
	Neutrophil count	−3.19	0.0018	−3.51	0.0006	-	ns	−6.18	3.22×10^−08^
Antigen- stimulated^G^	IL-1RA	3.26	0.0026	3.19	0.0033	-	ns	-	>0.05
	IL-12	3.20	0.0030	3.26	0.0028	-	ns	-	>0.05
	CCL11	-	ns	-	ns	-	ns	−7.82	6.45×10^−09^
	HGF	-	ns	-	ns	-	ns	−3.32	0.0023

A: age, sex, duration of symptoms pre-diagnosis, duration of antimicrobial therapy pre-sampling, baseline serum 25-hydroxyvitamin D concentration; B: MTB genotype categorised as Indo-Oceanic, East Asian, East African-Indian, Euro-American and West African strain lineage. C: host *DBP* genotype categorised as Gc1F/1F, 1F/2, 2/2, 1F/1S, 2/1S and 1S/1S as per ref [21]. D: age, sex, duration of symptoms pre-diagnosis, duration of antimicrobial therapy pre-sampling, isolate sensitive vs. resistant to isoniazid, allocation to vitamin D vs. placebo. E. t-statistic (regression co-efficient/standard deviation ) represents magnitude of difference between ethnic groups; a negative t-statistic indicates a lower concentration of immunological parameter in participants of African vs. Eurasian ancestry, and vice versa. F, p values derived using the t-test for general linear models, with adjustment for covariates (A–D). Parameters with a false discovery rate (q-value)>0.05, determined by the Benjamini Hochberg approach, were designated non-significant (ns). For the purposes of applying this approach, each ‘family’ of hypotheses corresponded to a single column of presented p values for either circulating or antigen-stimulated samples. G, stimulated with recombinant culture filtrate protein, 10 kDa (rCFP-10).

MTB, *Mycobacterium tuberculosis*; CCL, CC chemokine ligand; CXCL, CXC chemokine ligand; DBP, vitamin D binding protein; IL, interleukin; RA, receptor antagonist; HGF, hepatocyte growth factor.

**Figure 3:**
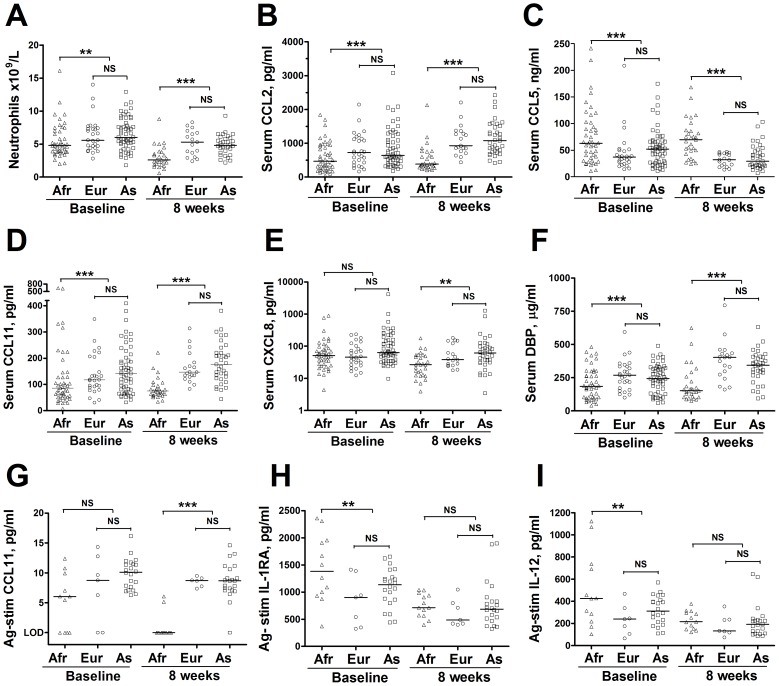
Inflammatory profiles of PTB patients of European/Middle Eastern and Central/South Asian ancestry are similar to each other, and different from those of patients of African ancestry. No statistically significant differences in neutrophil count (A), serum concentrations of CCL2 (B), CCL5 (C), CCL11 (D), CXCL8 (E), vitamin D binding protein (DBP) (F) or antigen-stimulated concentrations of CCL11 (G), IL1-RA (H) or IL-12 (I) were seen at baseline or at 8 weeks in PTB patients of European/Middle Eastern vs. Central/South Asian ancestry. However, when pooled data from patients of Eurasian ancestry were compared with those of patients of African ancestry, baseline differences attained statistical significance for all parameters except serum CXCL8 concentration (E) and antigen-stimulated CCL11 (G), and 8-week differences attained statistical significance for all parameters except antigen-stimulated IL1-RA (H) and IL-12 (I). P-values were derived from the t-test for general linear models, separately applied to baseline and 8-week samples, with adjustment for the following covariates: age, sex, months of symptoms pre-diagnosis, duration of antimicrobial therapy pre-sampling and either baseline vitamin D status (baseline samples) or isoniazid sensitivity and allocation to vitamin D vs. placebo (8-week samples). Parameters with a false discovery rate (q-value)>0.05, determined by the Benjamini Hochberg approach, were designated non-significant (ns). ***, p<0.001; **, p = 0.001 to <0.01. Lines at median. LOD, limit of detection. Afr, African ancestry; Eur, European/Middle Eastern ancestry; As, Central/South Asian ancestry.

Despite the relative homogeneity in genetic structure in populations of Asian and European ancestry [13], healthy Asians and Europeans have previously been reported to have differing inflammatory profiles that associate with increased risk of coronary heart disease [17]. In order to explore whether combining these groups concealed significant heterogeneity in inflammatory profile in patients with TB, we stratified the analysis above, subdividing the Eurasian group into European and Middle Eastern vs. Central and South Asian. The resultant PCA plot showed that inflammatory profiles of these two groups clustered together, and were separated from those of patients of African ancestry (Figure 2). In keeping with this observation, no significant differences in circulating concentrations of inflammatory mediators were found between Eurasian sub-groups (Figure 3). Our decision to combine data for Europeans and Asians in subsequent analyses was further justified by the finding that allele frequencies of two single nucleotide polymorphisms investigated in the *DBP* gene (rs 4588 and rs 7041) did not differ between participants of European/Middle Eastern vs. Central/South Asian ancestry (p≥0.32), but that they were different between Eurasians and Africans (p<0.001, Table 1)

In order to determine whether antigen-stimulated responses also differed between patients of African vs. Eurasian ancestry, whole blood samples taken from a sub-group of 42 patients (13 of African ancestry, and 29 of Eurasian ancestry) were stimulated *ex vivo* with the recombinant MTB antigen culture filtrate protein, 10 kDa (rCFP-10). The concentrations of 39 soluble factors listed in Table S1 were assayed in supernatants of whole blood samples taken at baseline and stimulated with rCFP-10 for 72 hours. The median concentrations of six soluble factors (IL-2, IL-5, IL-13, epidermal growth factor [EGF], FGF-β and MMP-7) were below the LOD at baseline and were excluded from further analyses; median values, ranges and LODs for these analytes are presented in Table S2. The remaining 33 parameters were analysed using the t-test for GLM with the same adjustment for covariates as conducted for circulating responses. Those that were different between groups were visualised by PCA. Two such parameters were found: antigen-stimulated concentrations of IL-1 receptor antagonist [IL-1RA] and IL-12 were both higher in participants of African vs. Eurasian ancestry (p≤0.0030; Table 2; Figure 1). As before, we conducted a sensitivity analysis to determine whether patients of European/Middle Eastern vs. Central/South Asian ancestry differed in their antigen-stimulated inflammatory profile: both the PCA plot (Figure 2) and scatter plots (Figure 3) showed similar patterns between these sub-groups. Moreover, conducting a t-test for GLM analysis did not identify any significant differences in inflammatory profile between the Eurasian sub-groups, further strengthening the rationale to pool data for patients of European/Middle Eastern and Central/South Asian ancestry together in subsequent analyses.

### Ethnic variation in inflammatory profile is not explained by variation in MTB strain lineage

MTB has co-evolved with humans, and different bacillary strains associate with different ethnic groups [2]; moreover, MTB strains of different lineage elicit differing immune responses *in vitro* [6]–[11] _ENREF_8. Ethnic variation in inflammatory profile in PTB might therefore be explained by differential representation of MTB strain lineages between ethnic groups. To investigate this possibility, genetic lineages of isolates from sputum of study participants were determined using multilocus Mycobacterial Interspersed Repetitive Units – Variable Number of Tandem Repeats (MIRU-VNTR) analysis [18], and frequencies of isolates of different lineage were compared between patient groups. Isolates of Indo-Oceanic (Lineage 1), East Asian (Lineage 2) and East African-Indian (Lineage 3) lineages tended to be under-represented, and isolates of Euro-American (Lineage 4) lineage over-represented, in participants of African vs. Eurasian ancestry (Table 1; p = 0.08). We therefore repeated the analyses of ethnic differences in inflammatory profile above, including additional statistical adjustment for MTB strain lineage: the set of parameters identified as being significantly different between patients of African vs. Eurasian ancestry was unchanged (Table 2), suggesting that MTB strain lineage was not a determinant of ethnic differences in inflammatory profile that we had observed.

**Figure 4:**
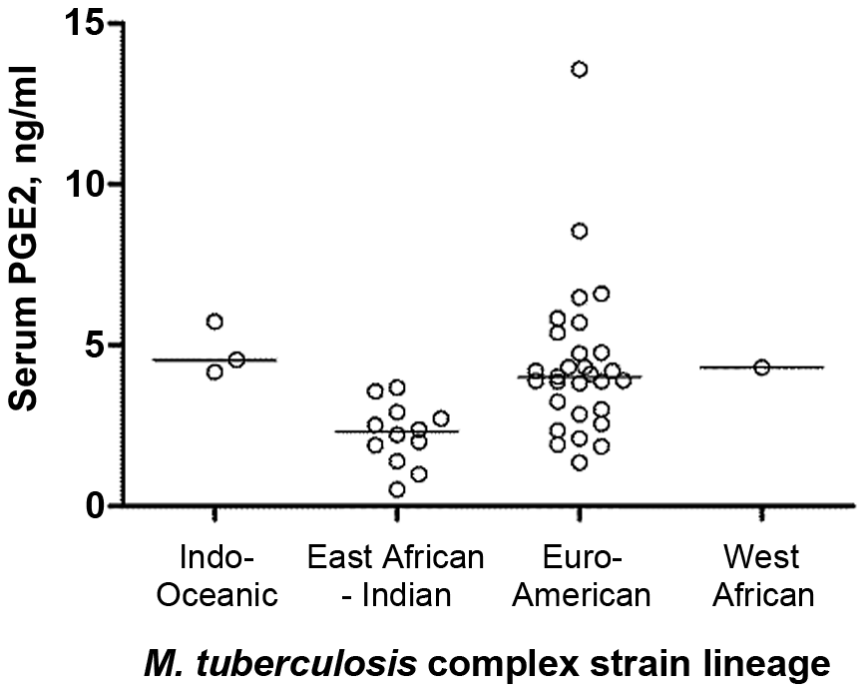
Baseline serum prostaglandin E2 (PGE2) concentration varies between PTB patients of African ancestry infected with different MTB strain lineages. Patients infected with isolates of East African-Indian lineage had lower PGE2 concentrations compared to those infected with isolates from other lineages (p = 0.0008, q = 0.0411). P-values were derived from the F-test for GLM, with adjustment for the following covariates: age, sex, months of symptoms pre-diagnosis, duration of antimicrobial therapy pre-sampling and baseline vitamin D status, with the false discovery rate (q-value) determined by the Benjamini Hochberg approach; Lines at median.

As a further test of the influence of MTB strain lineage on immune responses in the host, we conducted stratified analyses to compare inflammatory profiles associated with different MTB strain lineages in patients of African and Eurasian ancestry separately. Among patients of Eurasian ancestry, no statistically significant differences in either circulating or antigen-stimulated immune responses were observed between patients infected with organisms of different strain lineage. Among patients of African ancestry, serum concentrations of prostaglandin E2 (PGE2) were significantly lower in patients infected with MTB of East African-Indian lineage compared to those infected by other lineages (p = 0.0008; Figure 4), but no inter-lineage differences were seen for any other circulating parameter, or for any antigen-stimulated parameter investigated.

### Ethnic variation in host DBP genotype relates to ethnic differences in inflammatory profile

**Figure 5:**
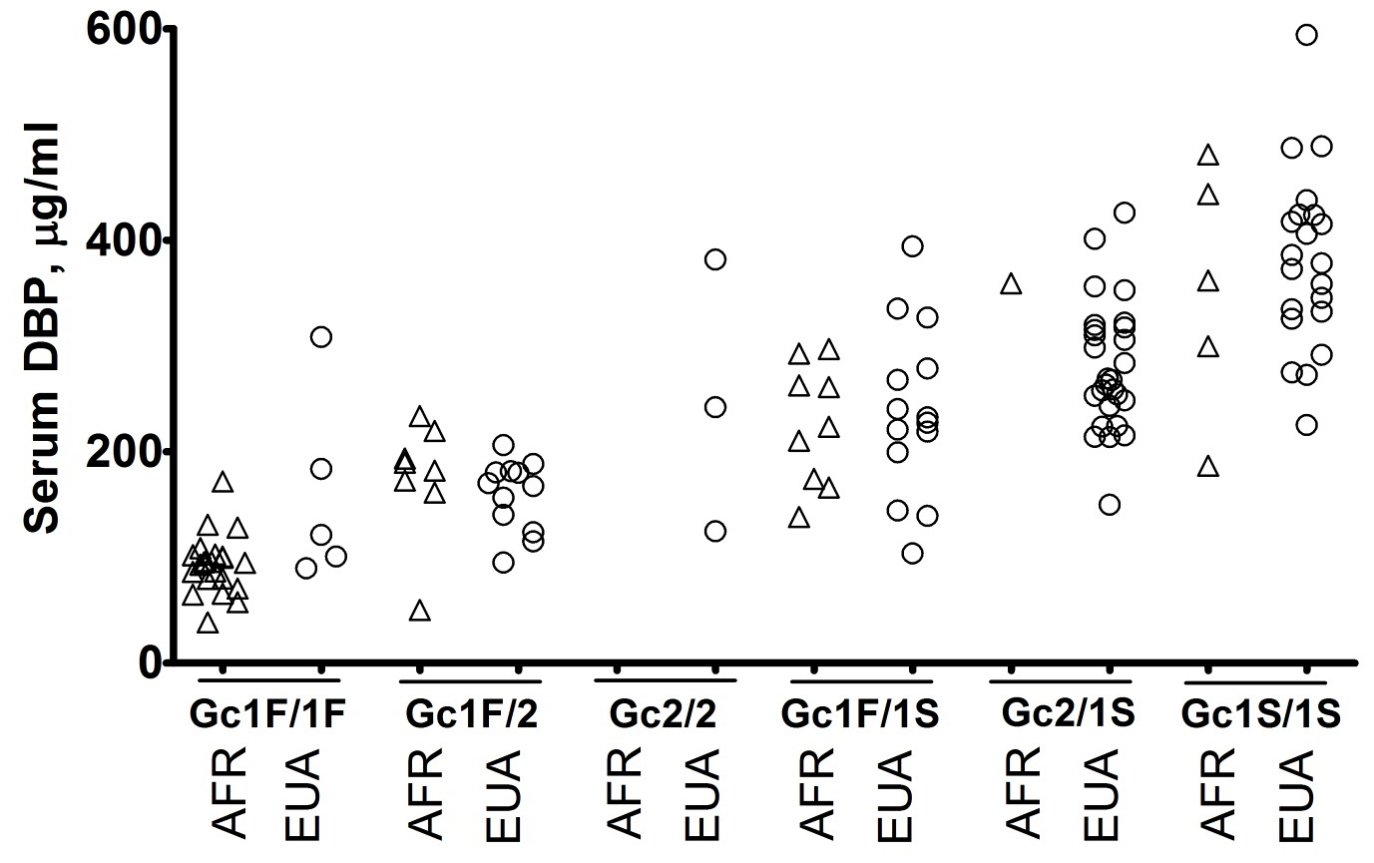
Serum vitamin D binding protein (DBP) concentration in patients with newly-diagnosed PTB by ***DBP*** **genotype and ethnic group.** DBP concentration varied with *DBP* genotype, with patients of Gc1F/1F genotype having the lowest concentrations, and those with Gc1S/1S genotype having the highest concentrations, irrespective of ethnic group (p<0.0001 for comparison by genotype with ethnic groups pooled; p>0.05 for ethnic comparison within each genotype). Kruskal-Wallis test with Dunn's multiple comparison test. AFR, African ancestry; EUA, Eurasian ancestry.

Since ethnic variation in the distribution of MTB strain lineages did not associate with differences in inflammatory profile observed between participants of African vs. Eurasian ancestry, we proceeded to investigate whether these differences might arise as a result of genetic variation in the host – a hypothesis suggested by results of human genome scans identifying chromosomal regions that influence immune responses to *M. tuberculosis* [19], [20]. To explore this possibility, we investigated two common functional single nucleotide polymorphisms in the *DBP* gene (rs4588 and rs7041), combinations of which form three haplotypes (Gc1F, Gc1S and Gc2). These polymorphisms were selected for investigation on the basis that they have been shown to influence antimycobacterial immune responses; that their frequency varies between people of African vs. Eurasian ancestry [21]; and that we had identified a significant difference in DBP concentration between ethnic groups. Rs4588 and rs7041 genotypes were determined, and haplotype frequencies were compared between ethnic groups: Gc1F carriers were over-represented, and Gc2 carriers under-represented, in patients of African vs. Eurasian ancestry (p<0.0001, Table 1). Moreover, serum DBP concentration in newly-diagnosed TB patients varied with *DBP* genotype, with those of Gc1F/1F genotype having the lowest concentrations and those with Gc1S/1S genotype having the highest concentrations, irrespective of ethnic group (p<0.0001 for comparison by genotype; p>0.05 for ethnic comparison within each genotype; Figure 5).

**Figure 6:**
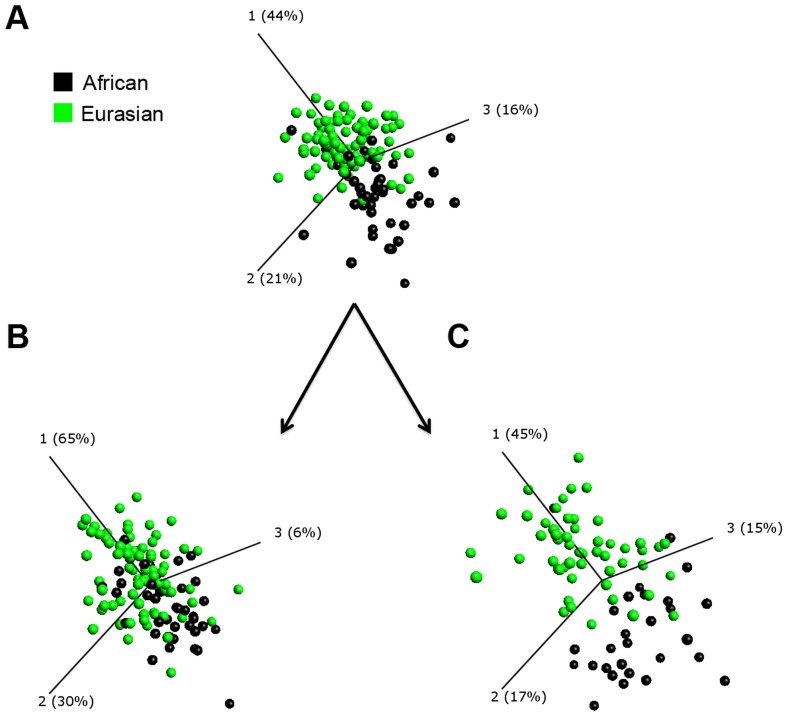
Ethnic variation in circulating inflammatory responses in PTB is reduced by adjustment for host vitamin D binding protein (***DBP*** **) genotype, but accentuated by administration of intensive-phase antimicrobial therapy.** Principal component analysis (PCA) plots generated using circulating parameters which contribute to ethnic variation in inflammatory profile in patients with PTB are displayed. Each point represents one patient, and its position in the plot is determined by the combined effects of all circulating parameters measured for that patient sample contributing significantly to ethnic variation in inflammatory profile. The distance between sample points represents Euclidean distance. The first 3 component vectors are displayed, along with a % figure signifying the proportion of the variability in the data that each component accounts for. A, PCA plot of circulating immunological parameters in patients of African (black, n = 45) *vs.* Eurasian (green, n = 83) ancestry at baseline. B, PCA plot as for A, but with additional statistical adjustment for host *DBP* genotype; separation of samples from patients of African *vs.* Eurasian ancestry is reduced as compared with panel A, indicating that *DBP* genotype accounts for a significant degree of ethnic variation in baseline inflammatory profile. C, PCA plot of circulating immunological parameters after intensive phase antimicrobial therapy; separation of samples from patients of African *vs.* Eurasian ancestry is increased as compared with panel A, indicating that ethnic differences in inflammatory profile become more marked following two months of treatment.

We therefore repeated the analysis of ethnic differences in inflammatory profiles, this time including statistical adjustment for *DBP* genotype in addition to the phenotypic characteristics previously incorporated in the model. Ethnic differences in neutrophil count, in serum DBP concentration, and in antigen-stimulated responses that had previously attained statistical significance in the original model were rendered non-significant by this adjustment (Table 2). The effect of the adjustment for *DBP* genotype is further illustrated in Figure 6, which shows a reduction in separation of samples from patients of African vs. Eurasian ancestry in a PCA plot after incorporation of *DBP* genotype in the model. We conclude that ethnic variation in *DBP* genotype associates with variation in inflammatory profiles observed between PTB patients of African vs. Eurasian ancestry.

### Ethnic variation in inflammatory profiles persists after completion of intensive-phase antituberculous therapy

We next proceeded to investigate whether ethnic differences in inflammatory profiles persisted after completion of intensive-phase antituberculous therapy. Concentrations of the same immunological parameters described above were assayed in samples of serum, plasma and whole blood taken after 8 weeks of antituberculous therapy from a cohort of 85 patients (30 of African ancestry and 55 of Eurasian ancestry) who fulfilled pre-defined criteria for inclusion in the per-protocol analysis of the clinical trial in which they were participating (Study Profile, Figure S1). Patients of different ethnic origin whose samples contributed to this analysis had similar demographic and clinical characteristics, the only statistically significant difference being a shorter duration of symptoms pre-diagnosis in patients of African vs. Eurasian ancestry (median 1.9 vs. 3.0 months respectively, p = 0.001). As before, parameters whose concentration was significantly different between participants of African vs. Eurasian ancestry were identified using the t-test for GLM, with adjustment for clinical and demographic covariates with potential to influence the effects of antimicrobial therapy on immune responses (age, sex, duration of symptoms pre-diagnosis, duration of antimicrobial therapy pre-sampling, isoniazid sensitivity vs. resistance, and allocation to vitamin D vs. placebo in trial). The effect of significant parameters was then assessed visually using PCA.

This analysis revealed that ethnic differences in neutrophil count and serum concentrations of CCL2, CCL5, CCL11 and DBP persisted at 8 weeks (p≤7.84×10^−6^, Figure 1) and that an additional parameter, serum C-X-C chemokine ligand 8 (CXCL8) concentration, was lower in participants of African vs. Eurasian ancestry at this time point (p = 0.0018; Table 2). PCA plots of circulating inflammatory parameters sampled at different time points show that samples from patients of African vs. Eurasian ancestry were more widely separated at 8 weeks compared to baseline (Figure 6), indicating that ethnic variation in circulating inflammatory profile was more marked at 8 weeks than at baseline. Ethnic variation in antigen-stimulated responses was also observed in 8-week samples, with supernatant concentrations of antigen-stimulated CCL11 and hepatic growth factor (HGF) being significantly lower in patients of African vs. Eurasian ancestry after completion of intensive-phase therapy (p≤0.0023; Table 2, Figure 1).

As an additional check to determine whether any of this variation could be attributed to the effects of adjunctive vitamin D supplementation, which we have previously shown to be immunomodulatory [22], we repeated the analyses above in the sub-group of 47 participants allocated to the placebo arm of the clinical trial in which they were participating. Near-identical results were obtained in this smaller cohort (Table S4, Figure S2), confirming that ethnic differences in 8-week inflammatory profile observed in the analysis of all participants did not arise as a result of confounding by differential allocation to vitamin D vs. placebo in patients of African vs. Eurasian ancestry.

### Ethnic variation in inflammatory profiles associated with fast vs. slow response to antituberculous therapy

Given that differences in immune response have been reported to associate with differences in microbiological clearance among patients with PTB [23]–[25], and that speed of sputum culture conversion has been reported to vary between TB patients of African vs. Eurasian ancestry [14], [15] we wished to determine whether ethnic differences in inflammatory response associated with variation in microbiological response to therapy. To this end, we compared time to sputum culture conversion between participants of African vs. Eurasian ancestry in our cohort, and found no significant difference (p = 0.41). Since ethnic differences in inflammatory profile persisted throughout intensive-phase treatment, we reasoned that profiles associated with fast *vs.* slow sputum culture conversion might therefore differ between ethnic groups. To test this hypothesis, we classified each participant for whom sputum culture conversion data were available (n = 82) according to their time to sputum culture conversion from positive to negative, denoting those with time greater than or equal to the median value of 37.25 days ‘slow converters’ (n = 41), and those with time less than this value ‘fast converters’ (n = 41). Clinical characteristics of patients having fast vs. slow sputum culture conversion, stratified by ethnicity, are compared in Table S3. Slow sputum culture conversion was associated with older age and higher baseline sputum bacillary load among Eurasians (p≤0.01); similar trends were seen among Africans (p≤0.45). We then compared inflammatory profiles measured during the course of intensive-phase therapy between fast and slow sputum culture converters for patients of African vs. Eurasian ancestry. The interaction analysis for ethnic group was conducted using rank regression on the interaction term ‘week of sampling*speed of sputum culture conversion’ with adjustment for the same covariates as for the analysis of 8-week samples above, plus week of sampling and subject ID. Baseline bacillary load was not adjusted for as this is likely to be a significant driver of differences in inflammatory profiles between fast vs. slow converters.

**Figure 7:**
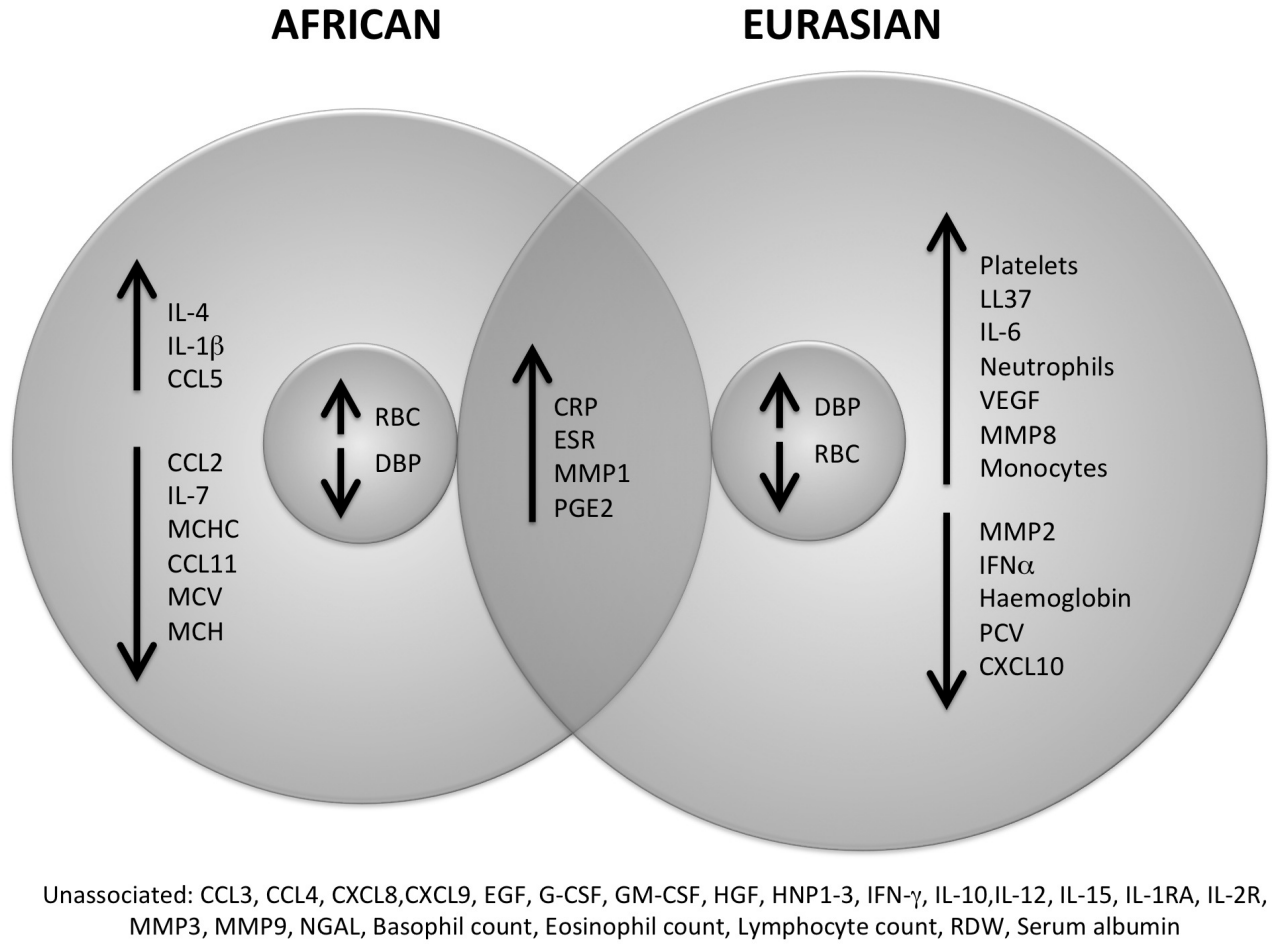
Circulating immunological correlates of slow sputum culture conversion differ between PTB patients of African vs. **Eurasian ancestry.** Upward arrows indicate parameters whose concentration was higher in patients with slow sputum culture conversion (defined as time to sputum culture conversion ≥37.25 days), and downward arrows indicate parameters whose concentration was lower in these patients. Only four of the 27 identified parameters have the same pattern of response in both ethnic groups.

**Figure 8:**
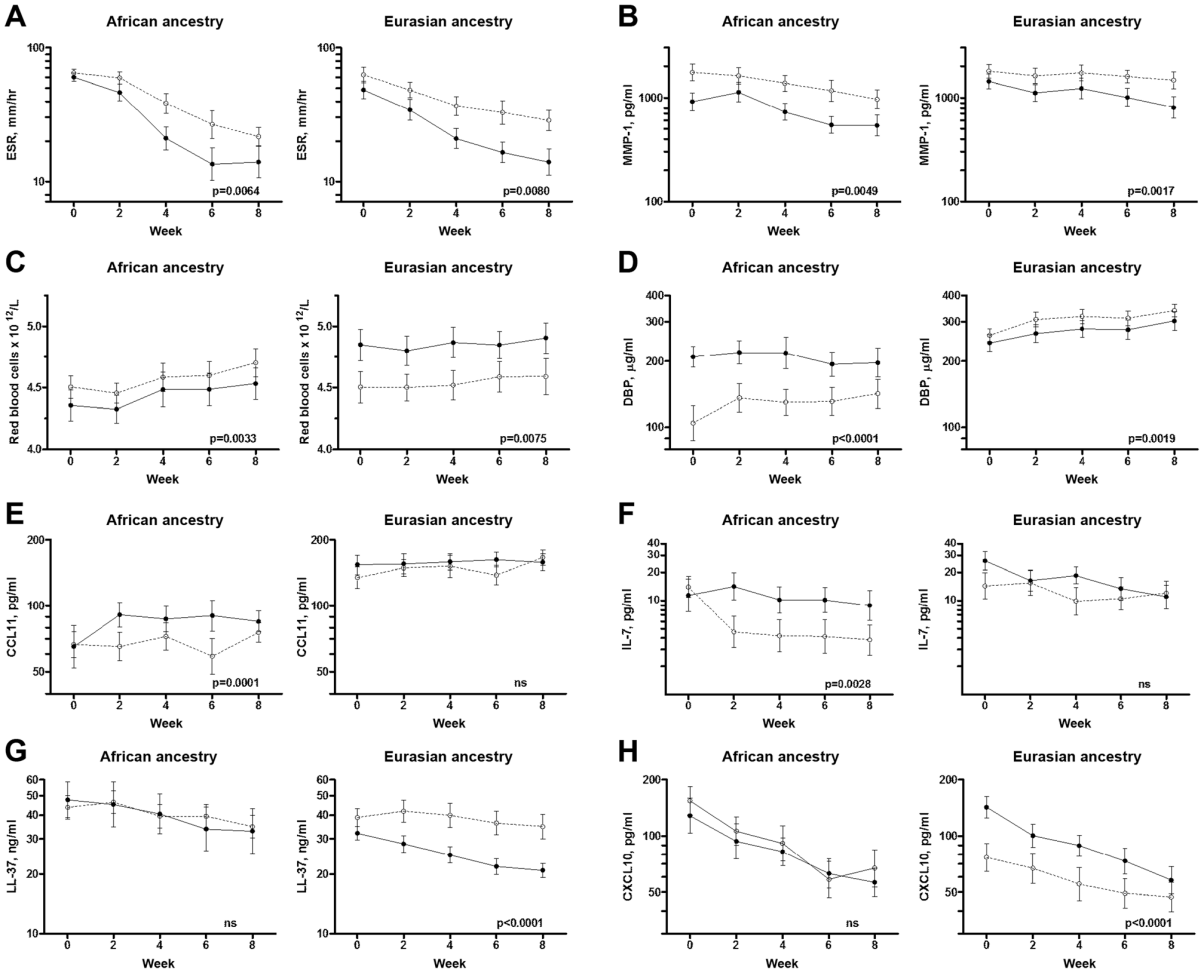
Circulating immunological correlates of time to sputum culture conversion in PTB patients of African vs. **Eurasian ancestry.** Higher ESR and serum MMP-1 concentration associated with slower sputum culture conversion in patients of African and Eurasian ancestry alike (A–B, p≤0.0080). Higher red blood cell count and lower serum vitamin D binding protein concentration associated with slower sputum culture conversion in patients of African ancestry but faster sputum culture conversion in those of Eurasian ancestry (C–D, p≤0.0075). Lower serum concentrations of CCL11 and IL-7 associated with slower sputum culture conversion in patients of African ancestry (p≤0.0028), but did not associate with treatment response in those of Eurasian ancestry (E–F). Higher plasma concentrations of cathelicidin LL-37 and lower serum concentration of CXCL10 associated with slower sputum culture conversion in patients of Eurasian ancestry (p≤4.98×10^−05^), but did not associate with treatment response in those of African ancestry (G–H). Fast (solid lines) vs. slow (dashed lines) sputum clearance defined as time to sputum culture conversion <37.25 days vs. ≥37.25 days, respectively. Means ± SEM at 0, 2, 4, 6 and 8 weeks of treatment are presented. Data for all parameters except red blood cell count were normalised by log_10_ transformation and the y-axis presented as 10∧(log value). P-values were generated using rank regression with covariates on the interaction term ‘week of sampling*speed of sputum culture conversion, with adjustment for the following covariates: age, sex, months of symptoms pre-diagnosis, duration of antimicrobial therapy pre-sampling, isoniazid sensitivity, allocation to vitamin D vs. placebo, week of sampling and subject ID. Parameters with a false discovery rate (q-value)>0.05, determined by the Benjamini Hochberg approach, were designated non-significant (ns).

The kinetics of circulating inflammatory responses differed markedly between patients with fast vs. slow sputum culture conversion, and immunological correlates of speed of sputum culture conversion differed between patients of African vs. Eurasian ancestry. Circulating immunological correlates of fast vs. slow sputum culture conversion in patients of African vs. Eurasian origin are summarised schematically in Figure 7, and presented in detail in Table 3 and Figure 8. Of the 50 parameters investigated, 27 were associated with speed of sputum culture conversion in one or both ethnic groups. Twelve of these parameters associated with speed of sputum conversion in patients of Eurasian ancestry only; nine associated with speed of sputum culture conversion in patients of African ancestry only; four associated similarly with speed of response in both ethnic groups; and two were differentially associated with speed of sputum culture conversion in patients of African vs. Eurasian ancestry, i.e. elevated levels of these markers associated with slower sputum culture conversion in one ethnic group and faster conversion in another.

**Table 3:** Circulating immunological correlates of speed of sputum conversion in PTB patients of African vs. Eurasian ancestry.

	African (n = 28)		Eurasian (n = 54)	
	t^A^	p^B^	t^A^	p^B^
IL-1β	2.76	0.0067	-	ns
IL-4	7.08	8.53×10^−11^	-	ns
IL-6	-	ns	4.08	6.09×10^−05^
IL-7	−3.05	0.0028	-	ns
IFN-α	-	ns	−2.94	0.0036
CXCL10	-	ns	−4.13	4.98×10^−05^
CCL2	−2.59	0.0108	-	ns
CCL5	2.60	0.0105	-	ns
CCL11	−3.97	0.0001	-	ns
VEGF	-	ns	3.34	0.0010
LL-37	-	ns	4.70	4.33×10^−06^
MMP-1	2.86	0.0049	3.17	0.0017
MMP-2	-	ns	−2.76	0.0062
MMP-8	-	ns	3.04	0.0026
PGE2	2.71	0.0077	3.03	0.0027
DBP	−5.00	1.86×10^−06^	3.13	0.0019
Hb	-	ns	−3.05	0.0026
MCV	−4.39	2.34×10^−05^	-	ns
PCV	-	ns	−3.26	0.0013
MCH	−5.20	7.62×10^−07^	-	ns
MCHC	−3.54	0.0006	-	ns
RBC	3.00	0.0033	−2.70	0.0075
Neutrophil count	-	ns	4.03	7.33×10^−05^
Monocyte count	-	ns	2.51	0.0127
Platelet count	-	ns	4.96	1.29×10^−06^
ESR	2.77	0.0064	2.67	0.0080
CRP	2.47	0.0149	4.62	6.18×10^−06^

A, t-statistic (regression co-efficient/standard deviation) represents magnitude of difference in immunological parameter between patients with fast vs. slow sputum culture conversion (<37.25 days vs. ≥37.25 days, respectively); a negative t-statistic indicates a lower concentration of immunological parameter in slower converters, and a positive t-statistic indicates a higher concentration of immunological parameter in slower converters. B, p values derived using rank regression with covariates on the interaction term ‘week of sampling*speed of sputum culture conversion’ and adjusting for age, sex, duration of symptoms pre-diagnosis, duration of antimicrobial therapy pre-sampling, isolate sensitive vs. resistant to isoniazid, allocation to vitamin D vs. placebo, week of sampling and subject ID. Parameters with a false discovery rate (q-value)>0.05, determined by the Benjamini Hochberg approach, were designated non-significant (ns). For the purposes of applying this approach, each ‘family’ of hypotheses corresponded to a single column of presented p values.

IL, interleukin; IFN, interferon; CXCL, CXC chemokine ligand; CCL, CC chemokine ligand; VEGF, vascular epidermal growth factor; LL-37, cathelicidin LL-37; MMP, matrix metalloproteinase; PG, prostaglandin; DBP, vitamin D binding protein; Hb, haemoglobin; MCV, mean corpuscular volume; PCV, packed cell volume; MCH, mean corpuscular haemoglobin; MCHC, mean corpuscular haemoglobin concentration; RBC, red blood cell count; ESR, erythrocyte sedimentation rate; CRP, C-reactive protein.

**Figure 9:**
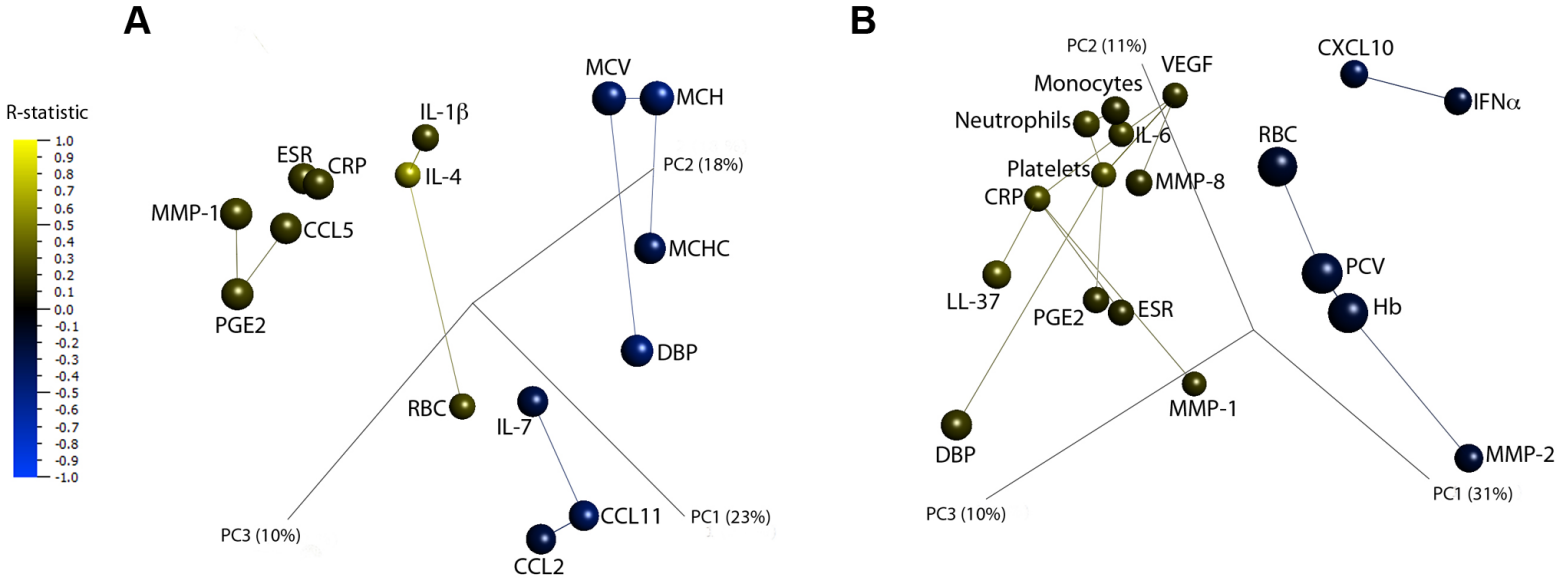
Three-dimensional principal component analysis plots depicting the correlation network of analytes significantly different between fast and slow culture converters of African and Eurasian ancestry during intensive phase anti-TB treatment. Plots for patients of African and Eurasian ancestry are shown in panels A and B, respectively. Individual points represent a single analyte. The distance between analytes is a measure of their Pearson correlation coefficients. Lines link analytes to their nearest neighbour, the analyte with the most similar pattern of change during the course of treatment. Each analyte is coloured according to its R-statistic value, calculated by rank regression interaction analysis. The concentration of analytes coloured blue is lower in patients with slow vs. fast sputum culture conversion, while the concentration of those coloured yellow is higher in patients with slow vs. fast sputum culture conversion. Component vectors are displayed, along with a % figure signifying the proportion of the variability in the data that each component accounts for.

As a further step to validate our findings, we applied network PCA to the parameters listed in Table 3 in order to investigate the relationship between changes in inflammatory parameters observed during treatment (Figure 9). For both ethnic groups, acute phase reactants erythrocyte sedimentation rate (ESR) and C-reactive protein (CRP) were linked and red cell parameters were linked to each other. In the African network MMP-1 was linked to its inducer PGE2, and chemokines CCL2 and CCL11 were closely linked, while in the Eurasian network, monocyte and neutrophil counts were closely linked. These linkages are consistent with biological understanding of the regulation of these inflammatory mediators and cell populations, validating results of the PCA. Kinetics of antigen-stimulated responses were not compared between groups due to small numbers within each sub-group.

## Discussion

Clinically significant ethnic differences in immune responses to *Plasmodium falciparum* and human immunodeficiency virus have previously been described [26], [27], but to our knowledge, this study is the first to address the question of whether inflammatory responses vary between TB patients of different ethnic origin. We report that inflammatory profiles vary significantly between TB patients of African and Eurasian ancestry having similar clinical and demographic characteristics, and that these differences associate primarily with ethnic variation in host rather than bacillary genotype. We also show that ethnic differences in inflammatory profiles observed at presentation persist after completion of intensive-phase therapy, and that immunological correlates of speed of sputum bacillary clearance differ markedly between patients of African vs. Eurasian ancestry. These findings have important implications for the design of studies investigating immunological biomarkers of response to antituberculous therapy.

African patients living in Africa have previously been reported to have more extensive disease at diagnosis than Europeans living in Europe, and to have lower rates of sputum conversion after intensive-phase antimicrobial therapy [14], [15], but controversy remains as to whether this reflects ethnic variation in host-pathogen interactions or geographical variation in laboratory practice and/or access to effective therapy. In our study – where patients of different ethnic origin were recruited in a single city, and where all microbiological samples were analysed in a single laboratory – we observed no difference in rates of cavitation or 2-month sputum culture conversion between patients of African vs. Eurasian ancestry. Despite this, we did observe significant ethnic variation in inflammatory profile between groups. Many of these differences were associated with variation in host *DBP* genotype, supporting the findings of an *in vitro* study reporting that DBP has broad influences on the antimycobacterial response [28]. This is plausible, given that this protein modulates macrophage activation and neutrophil chemotaxis, as well as performing its classical role in transport of vitamin D metabolites in the circulation [29]. We also observed ethnic variation in circulating and/or antigen-stimulated concentrations of cytokines (IL-1RA, IL-12) and chemokines (CCL2, CCL5, CCL11, CXCL8), many of which play key roles in the antimycobacterial immune response. The genes encoding these mediators are all polymorphic, and in some cases, ethnic variation in frequency of alleles influencing antimycobacterial responses has been reported [30], [31]. Study of functional associations of polymorphisms in these genes might yield insights into the genetic basis for ethnic variation in immune responses to MTB. Further investigation in other populations is also required to validate the ethnic differences in inflammatory profile that we report, as the large number of analyses and relatively modest sample size of our study could have led to Type I and Type II errors regarding specific parameters. Nevertheless, our main conclusions regarding strong ethnic group differences appear solid given the highly statistically significant differences found after stringent adjustment for multiple comparisons.

In contrast to the variation in inflammatory response between patients of different *DBP* genotype, relatively little difference in circulating and antigen-stimulated responses was seen between individuals infected with MTB strains of different lineage when multivariate analysis of the full cohort of 128 patients was performed. Secondary stratified analyses within the two main ethnic groups were conducted as a ‘belt and braces’ validation, to ensure that multivariate analysis had been successful in adjusting for potential ethnicity-related confounders of the relationship between MTB strain and immune profile. This secondary analysis identified only one analyte which was affected by lineage, and only in one ethnic group. The fact that the main analysis and the validation analyses yielded the same result - i.e. minimal effect of MTB strain on immune profile - lends considerable weight to our conclusion that MTB strain is not a major determinant of immune profile in tuberculosis.

This finding complements that of Pareek and colleagues, who recently reported that ethnicity is a powerful determinant of clinical TB phenotype independently of mycobacterial lineage [32]. Other investigators have reported that ‘modern’ strains elicit lower inflammatory responses than ‘ancient’ strains in macrophages, but that no difference in responses was seen in peripheral blood leukocytes [11], the population of cells investigated here. Further study is required to determine whether macrophages isolated from TB patients of different ethnic origin vary in their response to different MTB strains. Nevertheless, our observation that ethnic differences in inflammatory profile persisted after the several log-fold reduction in bacillary load induced by intensive-phase therapy tends to support the hypothesis that host, rather than bacillary, factors are the major determinants of ethnic variation in inflammatory profile. Such variation in inflammatory responses to antimicrobial treatment might reflect ethnic differences in allele frequency of polymorphisms of drug transporter genes that have been shown to associate with pharmacokinetic response to rifampicin [33]. However, our observations that sputum conversion rates were similar in patients of African vs. Eurasian ancestry, and that ethnic differences in inflammatory responses post-therapy were qualitative rather than quantitative, does not support this hypothesis. It is more plausible that, as at baseline, ethnic differences in inflammatory profile after treatment represent ethnic variation in alleles encoding components of the inflammatory response. Such variation may have arisen as a result of differences in selective pressures on the immune response between populations that remained in Africa vs. those that migrated out of the continent some 70,000 years ago [1].

Whatever the underlying reasons for these differences, our observation that immunological correlates of fast vs. slow sputum culture conversion differ between patients of African vs. Eurasian ancestry has practical implications for the design of studies to identify immunological correlates of response to intensive-phase antituberculous therapy. Studies evaluating candidate biomarkers published to date have been relatively small, and have tended to investigate fewer parameters in smaller numbers of patients of homogeneous ancestry than in the current study. Our finding that high CRP and ESR associate with slow sputum culture conversion is in keeping with other reports [25], [34]. Larger studies are now needed; our findings indicate that the validity of candidate biomarkers of treatment response identified by such studies will need to be evaluated in patients of different ancestry, as the inflammatory response in TB is ethnically heterogeneous.

## Materials and Methods

### Participants

The patients included in this study were participants in the AdjuVIT study - a double-blind randomised placebo-controlled trial of high-dose vitamin D during intensive-phase antimicrobial treatment of pulmonary TB, conducted in London, UK. Recruitment commenced on January 25^th^ 2007, and ended on July 3^rd^ 2009. A detailed account of study design has previously been given [12]. Participants self-defined their ethnic origin using the UK Office of National Statistics classification [35] and this information was used to attribute ancestry into one of five groups: African, Eurasian (incorporating participants of European, Middle Eastern, Central or South Asian ethnic origin), East Asian, Oceanic and American [13]. Baseline assessment included collection of a sputum sample for microscopy and culture and a blood sample. Fresh whole blood was sent for determination of full blood count and ESR and *ex vivo* stimulation with a mycobacterial antigen as described below. Aliquots of serum, plasma and whole blood were also stored at −80°C until completion of the trial. Participants were reviewed at 14, 28, 42 and 56 days after starting antituberculous therapy to assess clinical status and to monitor for adverse events. Blood and sputum samples were collected at each time-point and processed as above. Full characterisation of inflammatory profile was performed in the sub-set of participants who fulfilled pre-defined criteria for per-protocol analysis (i.e. those infected with a rifampicin-sensitive isolate of *M. tuberculosis* who received at least three doses of study preparation, who were compliant with antituberculous therapy, who did not take second-line antituberculous therapy or oral corticosteroids, who completed all study visits and who were not HIV sero-positive). The study was approved by East London and The City Research Ethics Committee (ref 06/Q0605/83), and registered with ClinicalTrials.gov (NCT00419068). Written informed consent was obtained from all participants before enrolment.

### Antigen-stimulated whole blood assay (WBA)

For all participants recruited on or after May 15^th^ 2008, fresh whole blood was diluted 1∶10 in RPMI 1640 medium (Sigma-Aldrich, Gillingham, UK) and duplicate 180 µl aliquots were stimulated in 96-well plates at 37°C in the presence of 5% CO_2_ with rCFP-10 (Rv3874, Proteix Biotechnologies, Vestek, Czech Republic; final concentration 2.5 µg/ml) or 2% bovine serum albumin in phosphate buffered saline (negative control). Plates were centrifuged after 72 hours' incubation, and cell-free supernatants were aspirated and frozen at −80°C pending immunological analysis. rCFP-10 was tested for presence of endotoxin: concentration was found to be 260 IU (EU)/mg, working concentration 63 pg/ml. Addition of this concentration of endotoxin to TB patients' whole blood in control experiments did not stimulate cytokine or chemokine secretion.

### Immunological analysis

Immunological parameters were selected on the basis that they played a role in host defence against MTB and/or that they were recognised biomarkers of disease activity [36]. Concentrations of 43 soluble factors listed in Table S1 were determined in serum/plasma as follows. Serum CRP and albumin concentrations were assayed using an Architect ci8200 analyser (Abbott Diagnostics, Chicago, IL, USA). Serum concentrations of IL-1β, IL-1RA, IL-2, IL-2R, IL-4, IL-5, IL-6, IL-7, IL-10, IL-12 (p40/p70), IL-13, IL-15, IL-17, G-CSF, GM-CSF, IFN-α, IFN-γ, TNF, CXCL8, CXCL9, CXCL10, CCL2, CCL3, CCL4, CCL5, CCL11, EGF, FGF-β, HGF and vascular endothelial growth factor (VEGF) were quantified using a human 30-plex bead immunoassay panel (sensitivity [sens.] according to Lot #617361, Invitrogen, Camarillo, CA, USA). Serum samples required high dilution for accurate determination of CCL5 concentration and all were re-assayed using a single-plex bead assay (Invitrogen). Serum PGE2 concentration was analysed by high sensitivity competitive enzyme immunoassay (EIA; Assay Designs, Ann Arbo37.25r, MI, USA; sens. 13.4 pg/ml). Plasma concentrations of antimicrobial peptides (AMP) LL-37 (sens. 31 pg/ml), HNP1-3 (sens. 156 pg/ml) and NGAL (sens. 400 pg/ml) were analysed by ELISA (Hycult Biotechnology, Uden, The Netherlands). Plasma concentrations of MMP-1, -2, -3, -7 and -8 were determined by Fluorokine MAP multianlalyte profiling (sens. according to Lot #273379, R&D systems); plasma concentration of MMP-9 was determined by DuoSet ELISA (sens. 3 pg/ml, R&D systems). Serum concentration of DBP was determined by ELISA (sens. 0.65 ng/ml, R&D systems). Multi-plex bead assays were performed on a Luminex 200 anlayzer (Luminex Corporation, Austin, TX, USA). ELISA and EIA absorbances were measured using a Benchmark Plus microplate spectrophotometer (Bio-Rad Laboratories, Hertfordshire, UK). The concentrations of 39 of these analytes (all of the above except DBP, PGE2, CRP and albumin; listed in Table S1) were also determined in WBA supernatants. Antigen-stimulated AMP and MMP concentrations were corrected by subtraction of unstimulated values. For MMP-2, -3, -8 and HNP and NGAL, unstimulated values were generally greater than stimulated values and this was the case sometimes for MMP-9 and LL-37. Cytokine/chemokine values were generally undetectable in unstimulated samples and a correction was not applied.

Fourteen haematological parameters listed in Table S1 were also measured in fresh whole blood. Full blood counts were performed using a LH750 haematology analyser (Beckman Coulter, Brea, CA, USA). ESR was measured by the Wintrobe method using a s2000 analyser (Desaga, Wiseloch, Germany).

### DNA extraction and genotyping

Human DNA was extracted from whole blood using the Promega Wizard SV 96 Genomic DNA Purification System on the Biomek FX robot (Beckman Coulter), quantified using the Nanodrop spectrophotometer and normalised to 5 ng/ml. 10 ng DNA was used as template for 5 ml pre-developed TaqMan assays (Applied Biosystems, Foster City, CA, USA) to type the *StyI* (rs4588) and *HaeIII* (rs7041) polymorphisms of the vitamin D binding protein. These assays were performed on the ABI 7900HT platform in 384-well format, and data were analysed with Autocaller software. *DBP* haplotypes were deduced from *StyI* and *HaeIII* genotypes as previously described [21]. Mycobacterial DNA was extracted and genotyped using automated 15 mycobacterial interspersed repetitive unit–VNTR as previously described [18].

### Statistical analysis

Contingency tables were analysed using chi-square tests, unless more than 20% of cells in a table had an expected frequency of <5, when Fisher's exact tests were employed. Median serum DBP concentration was compared between groups using a Kruskal-Wallis test with Dunn's post hoc test to correct for multiple comparisons. Time to sputum culture conversion was compared between groups using a logrank test. Analyte concentrations were calculated from raw luminex, ELISA and EIA data using Masterplex ReaderFit software (Hitachi Solutions America, San Francisco, CA, USA) and these calculated values were plotted using GraphPad Prism 5 software (La Jolla, CA, USA). Linear modelling and PCA was conducted using Qlucore Omics Explorer 2.2 software (Qlucore AB, Lund, Sweden). Analyte concentrations were log_2_ converted and the variance was normalized to 1. For analytes that were undetectable in at least one sample, the ‘limit of detection’ value was added to every measured value for that analyte prior to log_2_ conversion. Missing values were imputed by K nearest neighbours (k-NN) [37]: for circulating parameters, 2.5% of data points were missing; for CFP-10-stimulated parameters, 3% of data points were missing. Parameters whose concentration differed significantly between patients of African vs. Eurasian ancestry were identified using the t-test for GLM with adjustment for covariates with potential to influence the inflammatory profile using the eliminated factors approach. This fits a multiple regression model to all covariates, and subtracts the expression values predicted by this model from the observed values in order to remove covariate effects between patients [38]. An F-test for GLM with adjustment for covariates with potential to influence the inflammatory profile was performed to identify parameters whose concentration varied according to MTB strain lineage within each ethnic group. Parameters associating with slow vs. fast sputum culture conversion within each ethnic group were identified by rank regression analysis on the interaction term ‘week of sampling*speed of sputum culture conversion’ with adjustment for covariates with potential to influence the inflammatory profile, week of sampling (to correct for effects of treatment duration alone) and subject ID (to correct for repeated measures). Rank regression was conducted by replacing the ordinal interaction categorical predictors with numerical predictors, followed by a normal linear regression. Samples were ordered alternately fast, then slow, with increasing time since treatment initiation [39].

These analyses yield t-statistics (calculated as the regression co-efficient for each parameter divided by its standard deviation) representing the magnitude of difference in concentration of a given parameter between groups being compared; p values, representing the probability that such differences could have arisen by chance alone; and q values, which define the lowest false discovery rate (FDR) for which the hypothesis would be accepted under the Benjamini-Hochberg procedure for multiple testing correction [40]. Thresholds of 0.05 were applied for p and q values throughout.

PCA networks were created using one connection, i.e. by connecting each analyte to the other analyte that it shares the most similar pattern of change with over time; the distance between analytes in the network represents their Pearson correlation coefficients. Points in the network are coloured according to the value of the R-statistic generated for each analyte from the rank regression interaction analysis, which identified variables that had a significantly different pattern of change between slow and fast responders over time. The value of the R-statistic indicates the proportion of the total variation of that variable which is explained by the model tested. It is calculated as the square root of the *R^2^*-statistic, and the sign indicates the direction of the observed effect. A positive R-statistic indicates a higher concentration of that analyte in slow *vs.* fast culture converters, and vice versa.

## Supporting Information

Figure S1 **Study profile.**
(PPT)

Figure S2 **Ethnic variation in inflammatory profile after completion of intensive-phase antituberculous therapy: participants allocated to placebo only.** Patients of African ancestry had lower neutrophil counts (A), lower serum concentrations of CCL2, CCL11, CXCL8 and DBP (B–E) and lower levels of antigen-simulated CCL11 (F), but higher serum concentrations of CCL5 (G), than those of Eurasian ancestry. P-values were derived from t-tests for general linear models applied to 8-week placebo samples with adjustment for the following covariates: age, sex, months of symptoms pre-diagnosis, duration of antimicrobial therapy pre-sampling and isoniazid sensitivity. P-values are only indicated for parameters with a false discovery rate (q-value)≤0.05, as determined by the Benjamini Hochberg approach. *** p<0.001, ** p = 0.001 to <0.01. LOD, limit of detection. Lines at median.
(TIF)

Table S1 **Immunological parameters investigated.**
(DOCX)

Table S2 **Distribution of baseline concentrations of excluded analytes.**
(DOCX)

Table S3 **Characteristics of study participants entering analysis of correlates of treatment response.**
(DOCX)

Table S4 **Differences in inflammatory profile in PTB patients of African vs. Eurasian ancestry after completion of intensive-phase antimicrobial therapy: participants allocated to placebo arm only.**
(DOCX)

## References

[1] Gagneux S (2012) Host-pathogen coevolution in human tuberculosis. Philos Trans R Soc Lond B Biol Sci 367: 850–859. 22312052 10.1098/rstb.2011.0316 PMC3267123

[2] Hirsh AE, Tsolaki AG, DeRiemer K, Feldman MW, Small PM (2004) Stable association between strains of *Mycobacterium tuberculosis* and their human host populations. Proc Natl Acad Sci U S A 101: 4871–4876. 15041743 10.1073/pnas.0305627101 PMC387341

[3] Baker L, Brown T, Maiden MC, Drobniewski F (2004) Silent nucleotide polymorphisms and a phylogeny for *Mycobacterium tuberculosis* . Emerg Infect Dis 10: 1568–1577. 15498158 10.3201/eid1009.040046 PMC3320301

[4] Gagneux S, DeRiemer K, Van T, Kato-Maeda M, de Jong BC, et al (2006) Variable host-pathogen compatibility in *Mycobacterium tuberculosis* . Proc Natl Acad Sci U S A 103: 2869–2873. 16477032 10.1073/pnas.0511240103 PMC1413851

[5] Reed MB, Pichler VK, McIntosh F, Mattia A, Fallow A, et al (2009) Major *Mycobacterium tuberculosis* lineages associate with patient country of origin. J Clin Microbiol 47: 1119–1128. 19213699 10.1128/JCM.02142-08 PMC2668307

[6] Hoal-van Helden EG, Stanton LA, Warren R, Richardson M, van Helden PD (2001) Diversity of in vitro cytokine responses by human macrophages to infection by *Mycobacterium tuberculosis* strains. Cell Biol Int 25: 83–90. 11237411 10.1006/cbir.2000.0680

[7] Manca C, Reed MB, Freeman S, Mathema B, Kreiswirth B, et al (2004) Differential monocyte activation underlies strain-specific *Mycobacterium tuberculosis* pathogenesis. Infect Immun 72: 5511–5514. 15322056 10.1128/IAI.72.9.5511-5514.2004 PMC517425

[8] Theus SA, Cave MD, Eisenach KD (2005) Intracellular macrophage growth rates and cytokine profiles of *Mycobacterium tuberculosis* strains with different transmission dynamics. J Infect Dis 191: 453–460. 15633105 10.1086/425936

[9] Newton SM, Smith RJ, Wilkinson KA, Nicol MP, Garton NJ, et al (2006) A deletion defining a common Asian lineage of *Mycobacterium tuberculosis* associates with immune subversion. Proc Natl Acad Sci U S A 103: 15594–15598. 17028173 10.1073/pnas.0604283103 PMC1622867

[10] Tanveer M, Hasan Z, Kanji A, Hussain R, Hasan R (2009) Reduced TNF-alpha and IFN-gamma responses to Central Asian strain 1 and Beijing isolates of *Mycobacterium tuberculosis* in comparison with H37Rv strain. Trans R Soc Trop Med Hyg 103: 581–587. 19375139 10.1016/j.trstmh.2009.03.014

[11] Portevin D, Gagneux S, Comas I, Young D (2011) Human macrophage responses to clinical isolates from the *Mycobacterium tuberculosis* complex discriminate between ancient and modern lineages. PLoS Pathog 7: e1001307. 21408618 10.1371/journal.ppat.1001307 PMC3048359

[12] Martineau AR, Timms PM, Bothamley GH, Hanifa Y, Islam K, et al (2011) High-dose vitamin D_3_ during intensive-phase antimicrobial treatment of pulmonary tuberculosis: a double-blind randomised controlled trial. Lancet 377: 242–250. 21215445 10.1016/S0140-6736(10)61889-2 PMC4176755

[13] Rosenberg NA, Pritchard JK, Weber JL, Cann HM, Kidd KK, et al (2002) Genetic structure of human populations. Science 298: 2381–2385. 12493913 10.1126/science.1078311

[14] Fox W, Hutton PW, Sutherland I, Williams AW (1956) A comparison of acute extensive pulmonary tuberculosis and its response to chemotherapy in Britain and Uganda. Tubercle 37: 435–450. 13381029 10.1016/s0041-3879(56)80187-6

[15] Mac Kenzie WR, Heilig CM, Bozeman L, Johnson JL, Muzanye G, et al (2011) Geographic differences in time to culture conversion in liquid media: Tuberculosis Trials Consortium study 28. Culture conversion is delayed in Africa. PLoS ONE 6: e18358. 21494548 10.1371/journal.pone.0018358 PMC3073969

[16] Jolliffe IT (2002) Principal Component Analysis. New York: Springer-Verlag.

[17] Chambers JC, Eda S, Bassett P, Karim Y, Thompson SG, et al (2001) C-reactive protein, insulin resistance, central obesity, and coronary heart disease risk in Indian Asians from the United Kingdom compared with European whites. Circulation 104: 145–150. 11447077 10.1161/01.cir.104.2.145

[18] Brown T, Nikolayevskyy V, Velji P, Drobniewski F (2010) Associations between *Mycobacterium tuberculosis* strains and phenotypes. Emerg Infect Dis 16: 272–280. 20113558 10.3201/eid1602.091032 PMC2958017

[19] Wheeler E, Miller EN, Peacock CS, Donaldson IJ, Shaw MA, et al (2006) Genome-wide scan for loci influencing quantitative immune response traits in the Belem family study: comparison of methods and summary of results. Ann Hum Genet 70: 78–97. 16441259 10.1111/j.1529-8817.2005.00223.x

[20] Stein CM, Zalwango S, Malone LL, Won S, Mayanja-Kizza H, et al (2008) Genome scan of *M. tuberculosis* infection and disease in Ugandans. PLoS ONE 3: e4094. 19116662 10.1371/journal.pone.0004094 PMC2605555

[21] Martineau AR, Leandro AC, Anderson ST, Newton SM, Wilkinson KA, et al (2010) Association between Gc genotype and susceptibility to TB is dependent on vitamin D status. Eur Respir J 35: 1106–1112. 19797128 10.1183/09031936.00087009 PMC2864196

[22] Coussens AK, Wilkinson RJ, Hanifa Y, Nikolayevskyy V, Elkington PT, et al (2012) Vitamin D accelerates resolution of inflammatory responses during tuberculosis treatment. Proc Natl Acad Sci U S A 109: 15449–15454. 22949664 10.1073/pnas.1200072109 PMC3458393

[23] Brahmbhatt S, Black GF, Carroll NM, Beyers N, Salker F, et al (2006) Immune markers measured before treatment predict outcome of intensive phase tuberculosis therapy. Clin Exp Immunol 146: 243–252. 17034576 10.1111/j.1365-2249.2006.03211.x PMC1942062

[24] Djoba Siawaya JF, Bapela NB, Ronacher K, Beyers N, van Helden P, et al (2008) Differential expression of interleukin-4 (IL-4) and IL-4 delta 2 mRNA, but not transforming growth factor beta (TGF-beta), TGF-beta RII, Foxp3, gamma interferon, T-bet, or GATA-3 mRNA, in patients with fast and slow responses to antituberculosis treatment. Clin Vaccine Immunol 15: 1165–1170. 18579694 10.1128/CVI.00084-08 PMC2519297

[25] Djoba Siawaya JF, Bapela NB, Ronacher K, Veenstra H, Kidd M, et al (2008) Immune parameters as markers of tuberculosis extent of disease and early prediction of anti-tuberculosis chemotherapy response. J Infect 56: 340–347. 18359089 10.1016/j.jinf.2008.02.007

[26] Modiano D, Petrarca V, Sirima BS, Nebie I, Diallo D, et al (1996) Different response to *Plasmodium falciparum* malaria in west African sympatric ethnic groups. Proc Natl Acad Sci U S A 93: 13206–13211. 8917569 10.1073/pnas.93.23.13206 PMC24071

[27] Winkler C, An P, O'Brien SJ (2004) Patterns of ethnic diversity among the genes that influence AIDS. Hum Mol Genet 13 Spec No 1: R9–19. 10.1093/hmg/ddh075 14764621

[28] Chun RF, Lauridsen AL, Suon L, Zella LA, Pike JW, et al (2010) Vitamin D-binding protein directs monocyte responses to 25-hydroxy- and 1,25-dihydroxyvitamin D. J Clin Endocrinol Metab 95: 3368–3376. 20427486 10.1210/jc.2010-0195 PMC2928899

[29] Speeckaert M, Huang G, Delanghe JR, Taes YE (2006) Biological and clinical aspects of the vitamin D binding protein (Gc-globulin) and its polymorphism. Clin Chim Acta 372: 33–42. 16697362 10.1016/j.cca.2006.03.011

[30] Mwantembe O, Gaillard MC, Barkhuizen M, Pillay V, Berry SD, et al (2001) Ethnic differences in allelic associations of the interleukin-1 gene cluster in South African patients with inflammatory bowel disease (IBD) and in control individuals. Immunogenetics 52: 249–254. 11220627 10.1007/s002510000265

[31] Feng WX, Flores-Villanueva PO, Mokrousov I, Wu XR, Xiao J, et al (2012) CCL2–2518 (A/G) polymorphisms and tuberculosis susceptibility: a meta-analysis. Int J Tuberc Lung Dis 16: 150–156. 22137597 10.5588/ijtld.11.0205

[32] Pareek M, Evans J, Innes J, Smith G, Hingley-Wilson S, et al (2013) Ethnicity and mycobacterial lineage as determinants of tuberculosis disease phenotype. Thorax 68: 221–229. 23019255 10.1136/thoraxjnl-2012-201824 PMC5741171

[33] Weiner M, Peloquin C, Burman W, Luo CC, Engle M, et al (2010) Effects of tuberculosis, race, and human gene SLCO1B1 polymorphisms on rifampin concentrations. Antimicrob Agents Chemother 54: 4192–4200. 20660695 10.1128/AAC.00353-10 PMC2944564

[34] Dominguez-Castellano A, Muniain MA, Rodriguez-Bano J, Garcia M, Rios MJ, et al (2003) Factors associated with time to sputum smear conversion in active pulmonary tuberculosis. Int J Tuberc Lung Dis 7: 432–438. 12757043

[35] Office of National Statistics (2003) Ethnic group statistics: a guide for the collection and classification of ethnicity data. London: HMSO.

[36] Cooper AM (2009) Cell-mediated immune responses in tuberculosis. Annu Rev Immunol 27: 393–422. 19302046 10.1146/annurev.immunol.021908.132703 PMC4298253

[37] Troyanskaya O, Cantor M, Sherlock G, Brown P, Hastie T, et al (2001) Missing value estimation methods for DNA microarrays. Bioinformatics 17: 520–525. 11395428 10.1093/bioinformatics/17.6.520

[38] Wichura MJ (2006) The Coordinate-Free Approach to Linear Models. Cambridge: Cambridge University Press. Chapter 6, 110–136.

[39] McCullagh P (1980) Regression models for ordinal data (with discussion). J Roy Statist Soc B 42: 109–142.

[40] Benjamini Y, Hochberg Y (1995) Controlling the False Discovery Rate - a Practical and Powerful Approach to Multiple Testing. Journal of the Royal Statistical Society Series B-Methodological 57: 289–300.

