# Supplementary material for: Ethnic Variation in Inflammatory Profile in Tuberculosis
Source: PLoS Pathog. 2013 Jul 4;9(7):e1003468. doi: 10.1371/journal.ppat.1003468 (PMC3701709; doi:10.1371/journal.ppat.1003468)
Supplement: Figure S1 — Study profile. (PPT) [file ppat.1003468.s001.ppt]

## Slide 1
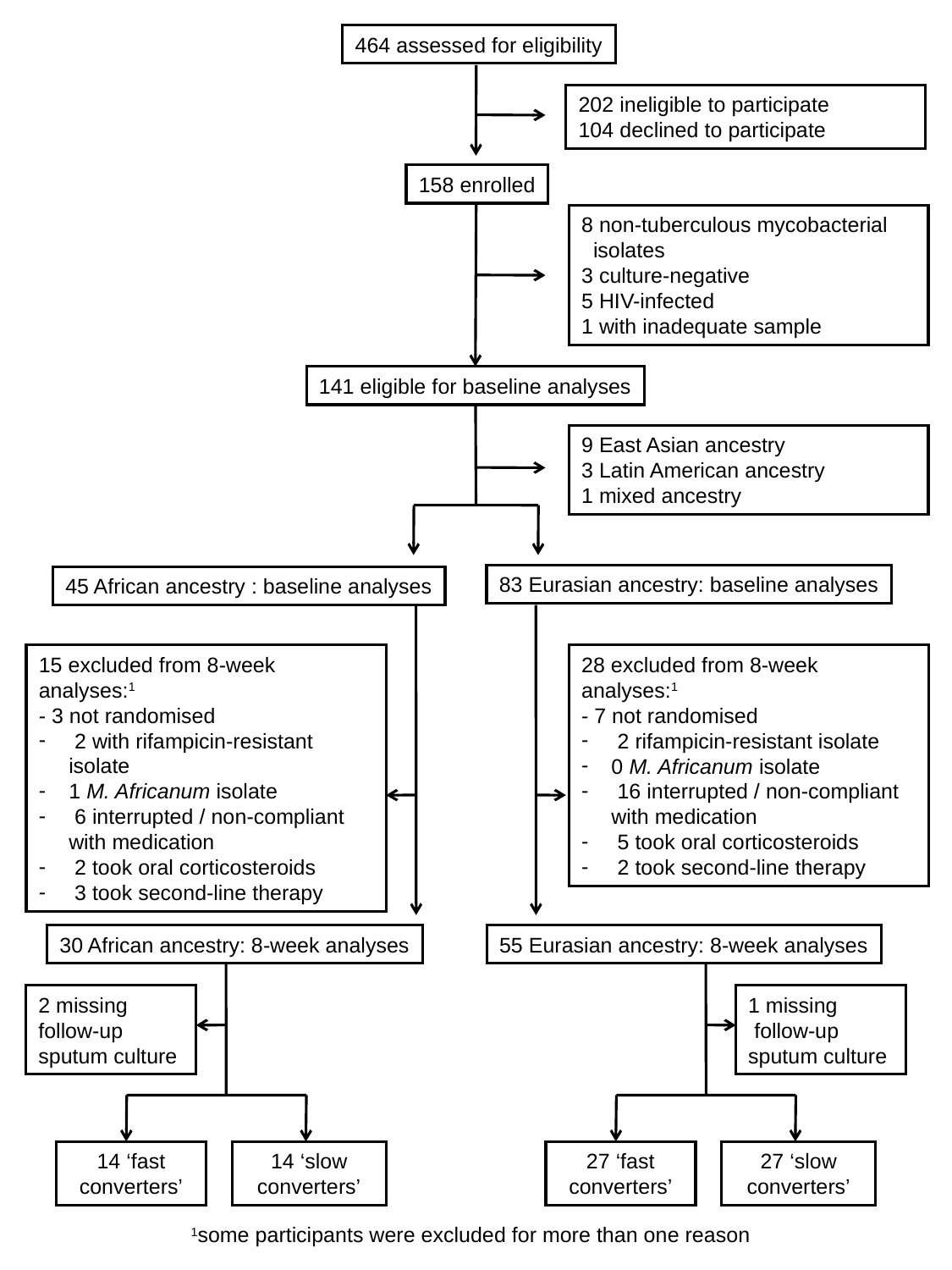

464 assessed for eligibility
202 ineligible to participate
104 declined to participate
158 enrolled
8 non-tuberculous mycobacterial isolates
3 culture-negative
5 HIV-infected
1 with inadequate sample
141 eligible for baseline analyses
9 East Asian ancestry
3 Latin American ancestry
1 mixed ancestry
83 Eurasian ancestry: baseline analyses
45 African ancestry : baseline analyses
15 excluded from 8-week analyses:1
- 3 not randomised
 2 with rifampicin-resistant isolate
1 M. Africanum isolate
 6 interrupted / non-compliant with medication
 2 took oral corticosteroids
 3 took second-line therapy
28 excluded from 8-week analyses:1
- 7 not randomised
 2 rifampicin-resistant isolate
0 M. Africanum isolate
 16 interrupted / non-compliant with medication
 5 took oral corticosteroids
 2 took second-line therapy
30 African ancestry: 8-week analyses
55 Eurasian ancestry: 8-week analyses
2 missing
follow-up
sputum culture
1 missing
 follow-up
sputum culture
14 ‘fast converters’
14 ‘slow converters’
27 ‘fast converters’
27 ‘slow converters’
1some participants were excluded for more than one reason
