# Supplementary material for: Ethnic Variation in Inflammatory Profile in Tuberculosis
Source: PLoS Pathog. 2013 Jul 4;9(7):e1003468. doi: 10.1371/journal.ppat.1003468 (PMC3701709; doi:10.1371/journal.ppat.1003468)
Supplement: Table S1 — Immunological parameters investigated. (DOCX) [file ppat.1003468.s003.docx]

**Table S1**

|  |  | **Immunological parameter (abbreviation)** | **Serum** | **Plasma** | **Whole blood** | **Supernatant**** |
| --- | --- | --- | --- | --- | --- | --- |
|  | |  |  |  |  |  |
| ***Soluble factors*** | |  |  |  |  |  |
|  | |  |  |  |  |  |
|  | *Cytokines* | Interleukin-1β (IL-1β) | X |  |  | X |
|  |  | Interleukin-1 receptor antagonist (IL-1RA) | X |  |  | X |
|  |  | Interleukin-2 (IL-2) | X* |  |  | X* |
|  |  | Interleukin-2 receptor (IL-2R) | X |  |  | X |
|  |  | Interleukin-4 (IL-4) | X |  |  | X |
|  |  | Interleukin-5 (IL-5) | X* |  |  | X* |
|  |  | Interleukin-6 (IL-6) | X |  |  | X |
|  |  | Interleukin-7 (IL-7) | X |  |  | X |
|  |  | Interleukin-10 (IL-10) | X |  |  | X |
|  |  | Interleukin-12 (IL-12) | X |  |  | X |
|  |  | Interleukin-13 (IL-13) | X* |  |  | X* |
|  |  | Interleukin-15 (IL-15) | X |  |  | X |
|  |  | Interleukin-17 (IL-17) | X* |  |  | X |
|  |  | Granulocyte colony-stimulating factor (G-CSF) | X |  |  | X |
|  |  | Granulocyte-macrophage colony-stimulating factor (GM-CSF) | X |  |  | X |
|  |  | Interferon-α (IFN-α) | X |  |  | X |
|  |  | Interferon-γ (IFN-γ) | X |  |  | X |
|  |  | Tumour necrosis factor (TNF) | X* |  |  | X |
|  | *Chemokines* | CXC chemokine ligand 8 (CXCL8) | X |  |  | X |
|  |  | CXC chemokine ligand 9 (CXCL9) | X |  |  | X |
|  |  | CXC chemokine ligand 10 (CXCL10) | X |  |  | X |
|  |  | CC chemokine ligand 2 (CCL2) | X |  |  | X |
|  |  | CC chemokine ligand 3 (CCL3) | X |  |  | X |
|  |  | CC chemokine ligand 4 (CCL4) | X |  |  | X |
|  |  | CC chemokine ligand 5 (CCL5) | X |  |  | X |
|  |  | CC chemokine ligand 11 (CCL11) | X |  |  | X |
|  | *Angiogenic factors* | Hepatocyte growth factor (HGF) | X |  |  | X |
|  |  | Epidermal growth factor (EGF) | X |  |  | X* |
|  |  | Basic fibroblast growth factor (FGF-β) | X* |  |  | X* |
|  |  | Vascular endothelial growth factor (VEGF) | X |  |  | X |
|  | *Antimicrobial peptides (AMP)* | Cathelicidin LL-37 (LL-37) |  | X |  | X |
|  |  | Neutrophil gelatinase-associated lipocalin (NGAL) |  | X |  | X |
|  |  | Human neutrophil peptides 1-3 (HNP1-3) |  | X |  | X |
|  | *Matrix Metalloproteinases (MMP)* | Matrix metalloproteinase-1 (MMP-1) |  | X |  | X |
|  |  | Matrix metalloproteinase-2 (MMP-2) |  | X |  | X |
|  |  | Matrix metalloproteinase-3 (MMP-3) |  | X |  | X |
|  |  | Matrix metalloproteinase-7 (MMP-7) |  | X* |  | X* |
|  |  | Matrix metalloproteinase-8 (MMP-8) |  | X |  | X |
|  |  | Matrix metalloproteinase-9 (MMP-9) |  | X |  | X |
|  | *Acute phase proteins* | C reactive protein (CRP) | X |  |  |  |
|  |  | Albumin | X |  |  |  |
|  | *Others* | Vitamin D binding protein (DBP) | X |  |  |  |
|  |  | Prostaglandin E2 (PGE2) | X |  |  |  |
|  |  |  |  |  |  |  |
| ***Haematological parameters*** | |  |  |  |  |  |
|  | |  |  |  |  |  |
|  | *Red blood cell parameters* | Haemoglobin (Hb) |  |  | X |  |
|  |  | Mean cell volume (MCV) |  |  | X |  |
|  |  | Packed cell volume (PCV) |  |  | X |  |
|  |  | Red blood cell distribution width (RDW) |  |  | X |  |
|  |  | Mean cell haemoglobin (MCH) |  |  | X |  |
|  |  | Mean corpuscular haemoglobin concentration (MCHC) |  |  | X |  |
|  |  | Red blood cell count (RBC) |  |  | X |  |
|  | *White blood cell parameters* | Neutrophil count |  |  | X |  |
|  |  | Lymphocyte count |  |  | X |  |
|  |  | Monocyte count |  |  | X |  |
|  |  | Eosinophil count |  |  | X |  |
|  |  | Basophil count |  |  | X |  |
|  | *Others* | Platelet count |  |  | X |  |
|  |  | Erythrocyte sedimentation rate (ESR) |  |  | X |  |

* indicates analytes whose median concentration at baseline was below the limit of detection in clinical samples; **72-hour supernatants of antigen-stimulated whole blood.
