# Supplementary material for: Ethnic Variation in Inflammatory Profile in Tuberculosis
Source: PLoS Pathog. 2013 Jul 4;9(7):e1003468. doi: 10.1371/journal.ppat.1003468 (PMC3701709; doi:10.1371/journal.ppat.1003468)
Supplement: Table S2 — Distribution of baseline concentrations of excluded analytes. (DOCX) [file ppat.1003468.s004.docx]

**Table S2**

|  | Median concentration,  pg/ml | Inter-quartile range,  pg/ml | Absolute range,  pg/ml | Limit of detection,  pg/ml |
| --- | --- | --- | --- | --- |
|  |  |  |  |  |
|  |  |  |  |  |
| Circulating analytes |  |  |  |  |
|  |  |  |  |  |
| MMP-7 | 897.6 | 531.0 to 1458.0 | 0.0 to 5537.2 | 940.0^A^ |
| FGF-β | 19.0 | 6.2 to 33.8 | 0.0 to 1030.3 | 22.0 |
| IL-13 | 31.5 | 20.2 to 40.4 | 0.0 to 316.5 | 36.0 |
| IL-5 | 0.5 | 0.2 to 2.3 | 0.0 to 59.8 | 3.0 |
| TNF | 2.2 | 1.0 to 4.1 | 0.0 to 27.8 | 5.0 |
| IL-2 | 2.2 | 0.5 to 6.1 | 0.0 to 153.1 | 6.0 |
|  |  |  |  |  |
| Antigen-stimulated analytes |  |  |  |  |
|  |  |  |  |  |
| MMP-7 | 77.9 | 68.3 to 114.8 | 0 to 590.2 | 376.0 ^A^ |
| FGF-β | 15.4 | 12.1 to 19.2 | 6.8 to 26.4 | 22.0 |
| IL-13 | 6.1 | 3.6 to 9.0 | 0.0 to 20.1 | 36.0 |
| IL-5 | 2.8 | 2.4 to 3.6 | 0.0 to 6.2 | 3.0 |
| IL-2 | 4.7 | 3.1 to 6.5 | 0.2 to 12.5 | 6.0 |
| EGF | 4.3 | 2.8 to 5.2 | 0.4 to 9.4 | 20.0 |

A, Limits of detection for circulating and antigen-stimulated concentrations of MMP-7 are different due to the use of a different dilution factor for the two sample types.

MMP, matrix metalloproteinase; FGF-β, basic fibroblast growth factor; IL, interleukin; TNF, tumour necrosis factor; EGF, epidermal growth factor.
