# Supplementary material for: Ethnic Variation in Inflammatory Profile in Tuberculosis
Source: PLoS Pathog. 2013 Jul 4;9(7):e1003468. doi: 10.1371/journal.ppat.1003468 (PMC3701709; doi:10.1371/journal.ppat.1003468)
Supplement: Table S3 — Characteristics of study participants entering analysis of correlates of treatment response. (DOCX) [file ppat.1003468.s005.docx]

**Table S3**

|  | **African,**  **all (n=28)** | **Eurasian,**  **all (n=54)** | **p** |  | **African, slow converters (n=14)** | **African, fast converters (n=14)** | **p** |  | **Eurasian, slow converters (n=27)** | **Eurasian, fast converters (n=27)** | **p** |
| --- | --- | --- | --- | --- | --- | --- | --- | --- | --- | --- | --- |
|  |  |  |  |  |  |  |  |  |  |  |  |
| Median age, years (IQR) | 31.5 (22.3 to 39.2) | 31.1 (24.8 to 40.2) | 0.63 |  | 34.1 (28.5 to 41.8) | 27.8 (20.4 to 37.3) | 0.25 |  | 36.6 (29.4 to 46.3) | 26.8 (23.7 to 34.0) | 0.003 |
| Sex |  |  |  |  |  |  |  |  |  |  |  |
| Male | 20 (71) | 44 (81) | 0.30 |  | 11 (79) | 9 (64) | 0.68 |  | 21 (78) | 23 (85) | 0.48 |
| Female | 8 (29) | 10 (19) |  |  | 3 (21) | 5 (36) |  |  | 6 (22) | 4 (15) |  |
| 8-week BMI, kg/m^2^ | 20.0 (19.1 to 21.6) | 20.2 (18.4 to 21.9) | 0.88 |  | 19.9 (19.2 to 21.6) | 20.2 (18.4 to 21.8) | 0.89 |  | 20.2 (17.4 to 21.9) | 20.3 (19.6 to 22.0) | 0.66 |
| Educated beyond 18 years | 17 (61) | 25 (46) | 0.25 |  | 8 (57) | 9 (64) | 0.70 |  | 9 (33) | 16 (59) | 0.06 |
| In UK before age 18 years | 11 (39) | 16 (30) | 0.46 |  | 3 (21) | 8 (57) | 0.12 |  | 8 (30) | 8 (30) | >0.99 |
| Occupation |  |  |  |  |  |  |  |  |  |  |  |
| Student | 7 (25) | 7 (13) | >0.99 |  | 2 (14) | 5 (36) | >0.99 |  | 0 (0) | 7 (26) | >0.99 |
| Employed | 19 (68) | 41 (76) |  |  | 10 (71) | 9 (64) |  |  | 23 (85) | 18 (66) |  |
| Unemployed | 2 (7) | 6 (11) |  |  | 2 (14) | 0 (0) |  |  | 4 (15) | 2 (7) |  |
| Diabetes mellitus | 1 (4) | 3 (6) | >0.99 |  | 1 (7) | 0 (0) | >0.99 |  | 2 (7) | 1 (4) | >0.99 |
| Median duration of symptoms pre-diagnosis, months (IQR) | 1.9 (1.0 to 2.9) | 3.0 (2.0 to 4.0) | 0.001 |  | 1.9 (1.0 to 3.0) | 1.5 (0.9 to 2.3) | 0.46 |  | 3.0 (2.0 to 5.0) | 3.0 (2.0 to 4.0) | 0.99 |
| Median duration of treatment pre-enrolment, days (IQR) | 2.0 (0.0 to 4.0) | 1.5 (0.0 to 3.0) | 0.48 |  | 2.0 (0.0 to 3.0) | 3.0 (0.0 to 5.0) | 0.37 |  | 2.0 (1.0 to 3.0) | 1.0 (0.0 to 2.0) | 0.07 |
| Baseline sputum smear |  |  |  |  |  |  |  |  |  |  |  |
| ≤3 AFB per high-power field | 13 (46) | 25 (46) | >0.99 |  | 5 (36) | 8 (57) | 0.45 |  | 8 (30) | 17 (63) | 0.01 |
| >3 AFB per high-power field | 15 (54) | 29 (54) |  |  | 9 (64) | 6 (43) |  |  | 19 (70) | 10 (37) |  |
| Baseline chest radiograph |  |  |  |  |  |  |  |  |  |  |  |
| Cavities present | 15 (54) | 35 (65) | 0.35 |  | 7 (50) | 6 (43) | >0.99 |  | 18 (67) | 17 (63) |  |
| Median no. zones affected, IQR | 2.0 (2.0 to 3.5) | 3.0 (2.0 to 4.0) | 0.39 |  | 2.0 (2.0 to 4.0) | 2.0 (1.0 to 3.3) | 0.30 |  | 3.0 (2.0 to 4.5) | 2.0 (2.0 to 4.0) | 0.48 |
| Drug sensitivity of isolate |  |  |  |  |  |  |  |  |  |  |  |
| Isoniazid-sensitive | 27 (96) | 49 (91) | 0.66 |  | 14 (100) | 13 (93) | >0.99 |  | 24 (89) | 25 (93) | >0.99 |
| Isoniazid-resistant | 1 (4) | 5 (9) |  |  | 0 (0) | 1 (7) |  |  | 3 (11) | 2 (7) |  |
| Allocation |  |  |  |  |  |  |  |  |  |  |  |
| Placebo | 19 (68) | 25 (46) | 0.10 |  | 9 (64) | 10 (71) | >0.99 |  | 15 (56) | 10 (37) | 0.17 |
| Vitamin D | 9 (32) | 29 (54) |  |  | 5 (36) | 4 (29) |  |  | 12 (44) | 17 (63) |  |

Data are number (%) or mean (standard deviation) except where stated. ‘Slow converters’ are defined as participants with time to sputum culture conversion ≥ 37.25 days; ‘fast converters’ are defined as those with time to sputum culture conversion < 37.25 days. IQR, inter-quartile range; BMI, body mass index. AFB, acid-fast bacilli.
