# Supplementary material for: Ethnic Variation in Inflammatory Profile in Tuberculosis
Source: PLoS Pathog. 2013 Jul 4;9(7):e1003468. doi: 10.1371/journal.ppat.1003468 (PMC3701709; doi:10.1371/journal.ppat.1003468)
Supplement: Table S4 — Differences in inflammatory profile in PTB patients of African vs. Eurasian ancestry after completion of intensive-phase antimicrobial therapy: participants allocated to placebo arm only. (DOCX) [file ppat.1003468.s006.docx]

**Table S4**

|  |  | **t**^A^ | **p**^B^ |
| --- | --- | --- | --- |
|  |  |  |  |
|  |  |  |  |
| Circulating | CCL2 | -3.46 | 0.0013 |
|  | CCL5 | 3.63 | 0.0008 |
|  | CCL11 | -5.06 | 1.11 x 10^-05^ |
|  | CXCL8 | -3.32 | 0.0020 |
|  | DBP | -6.10 | 4.12 x 10^-07^ |
|  | Neutrophil count | -4.58 | 4.88x 10^-05^ |
|  |  |  |  |
| Antigen-  stimulated^C^ | CCL11 | -7.31 | 1.21 x10^-06^ |
|  |  |  |  |

A. t-statistic (regression co-efficient / standard deviation) represents magnitude of difference between ethnic groups; a negative t-statistic indicates a lower concentration of immunological parameter in participants of African vs. Eurasian ancestry, and vice versa. B, p values derived using the t-test for general linear models with adjustment for the following covariates: age, sex, duration of symptoms pre-diagnosis, duration of antimicrobial therapy pre-sampling, isolate sensitive vs. resistant to isoniazid. C, stimulated with recombinant culture filtrate protein, 10 kDa (rCFP-10).

CCL, CC chemokine ligand; CXCL, CXC chemokine ligand; DBP, vitamin D binding protein.
